# Non-homogeneous combination of two porous genomes induces complex body shape trajectories in cyprinid hybrids

**DOI:** 10.1186/1742-9994-10-22

**Published:** 2013-05-01

**Authors:** Melthide Sinama, André Gilles, Caroline Costedoat, Emmanuel Corse, Jean-Michel Olivier, Rémi Chappaz, Nicolas Pech

**Affiliations:** 1 Aix-Marseille Université, CNRS, IRD, IMBE – UMR 7263, Equipe Evolution Génome Environnement, Centre Saint-Charles, Case 36, 3 place Victor Hugo, Marseille Cedex 3 13331, France; 2 Université Lyon 1, CNRS, UMR 5023 - LEHNA, Laboratoire d'Ecologie des Hydrosystèmes Naturels et Anthropisés Bât. Forel, Villeurbanne Cedex F-69622, France

**Keywords:** Hybridization, Introgression, Mitochondrial DNA, Microsatellites, Transgressive segregation, Ecological cline, Ontogeny, Fish, Invasive species

## Abstract

**Introduction:**

Hybridization is a common phenomenon in fish and is considered to be a major source of diversification. Deciphering the remoulding of genomic regions and phenotypes in zones where hybrid specimens occur is of particular interest to elucidate the emergence of evolutionary novelties. This approach is particularly challenging because the first step of hybridization seems to be the most important stage in the emergence of hybrid lineages. However, the signal can be significantly altered after only a few generations.

**Results:**

We studied 41 microsatellites and partial *cytochrome b* gene sequences in 970 specimens belonging to two fish species (*Chondrostoma nasus* and *Parachondrostoma toxostoma*) in allopatric/parapatric zones, hybrids between them in a natural sympatric zone: the Ardèche basin. We showed that the genomic architecture in hybrids presented pattern heterogeneity of selection for the different loci. Indeed, the upstream part of the river (Rosières and Labeaume) presented an overdominant fitness of heterozygotes (12.20%) corresponding to a genomic compatibility, and underselection was observed for 4.88%-7.32% of the loci tested indicating a genomic incompatibility. Moreover the upstream station (Rosières) presented a positive selection of invasive *C. nasus* homozygotes (17.07% to 21.95%) indicating that hybridization may increase the fitness of admixed individuals.

We showed that hybrid morphology (body shape based on 21 landmarks) correlated with genomic dilution indicating a species fingerprint. However, we demonstrated that the hybrid morphology was not a linear modification between the two parental species but a trade-off between several correlated traits.

**Conclusions:**

Hybrid specimens present a mosaic of genomic combination, showing regions with genomic compatibility and others with genomic incompatibility between the two species. Positive selection (invasive advantage ranging from 9.76% to 21.95% of the loci) was evidenced in the upstream part of the Ardèche indicating that environmental selection makes a substantial contribution. Although the presence of a dam is known to impose heterogeneous hybrid zones between these two species, we demonstrated in this study that a natural environment can also generate a hybrid zone with a large number (and diversity) of hybrids. The combination of the two genomes in the hybrids results in complex ontogenetic trajectories (with different morphological traits evolving at different rates) that correspond to novel developmental pathways.

## Introduction

One of the most important issues in evolutionary biology is the genetic basis of evolutionary novelties [1,2], and it is now clear that hybridization phenomena could induce significant contribution [3]. Indeed, hybridization is a common phenomenon in animals in general [4,5] and in fish in particular [6-10] but the part played by such phenomenon in the emergence of new genomic architectures and phenotypes in admixed individuals remains unclear [11].

Hybridization between divergent gene pools generates recombinant individuals. Analysis of such individuals provides information about genomic architecture associated with reproductive isolation between populations or species [12]. Both genetic factors contributing to genomic compatibility (endogenous selection) and environmental factors sorting specimens based on their fitness (exogenous selection) apply in zones where hybridization occurs naturally (called hybrid zones hereafter). The remoulding of genomic regions in hybrid zones may serve as a source of evolutionary novelties and these zones behave as biodiversity reactors generating waves of new genotypes. Selection may thus act on a vast number of genomic combinations from parental species to different hybrid genotypes; such hybrid genotypes may also serve as intermediaries for the transfer of adaptive genetic variation between parental populations [13-15]. Moreover, some authors have demonstrated that this phenomenon is possible in the early stages of the hybridization process [16-19].

Mathematical models are increasingly used to study the complexity of such biological phenomena. As reviewed in Payseur [20], most models used to analyse hybrid zones describe the relationship between allele frequency and geography. The clines in hybrid zones take a sigmoid shape to model the transition between species gradients. Several variables are used in these models (reviewed in [21]): the migration of the two species along the zone where clinal variation is observed (i.e. diffusion flattens the cline over space), the strength of selection (that maintains the sigmoid form) and the geographical transect of the observed alleles. Nolte *et al.*[16] pointed out that the analyses of such phenomenon need diagnostic loci to allow the two parental populations to be distinguished, and that the exclusion of loci that are not diagnostic can remove a part of the species polymorphism from the analysis.

Another approach is to use hybrid ancestries across the genome to predict introgression at individual loci. Pritchard *et al.*[22] developed a model-based clustering method for using multilocus data to infer population structure and assign individuals to populations. An alternative approach is based on multinomial logistic modelling [23,24]. The aim is to estimate the genomic cline as the genotype frequency at individual loci along a genomic admixture gradient (hybrid index = h [25]). A statistical test has been developed to identify markers that deviate from expectation (neutrality) based on a genome-wide admixture, and that can handle potential locus-specific selection [24].

Currently, it is not clear which evolutionary forces constrain the genomic architecture generated by hybridization process such that it “stabilizes” a new lineage. One of the best known examples in fish is that of the invasive hybrid lineages of *Cottus* in Europe. Nolte *et al.*[16] studied two hybrid zones involving *Cottus perifretum* and *Cottus rhenanus* using the genomic cline approach. Although the two species share similar overall genomic compositions, the observed patterns at individual loci differed substantially between zones indicating differences in external selection pressures or cryptic genetic differentiation of distinct parental populations. Recently, Stemshorn *et al.*[18] identified three distinct hybrid lineages, which have emerged out of a situation of secondary contact between *C. rhenanus* and *C. perifretum*. The examination of partially isolated lineages, such as invasive hybrid sculpins, may allow early adaptive genetic changes to be identified before they become confounded by differences arising due to speciation process.

Another particularity in hybrid zones concerns phenotypes of recombinant individuals. In the *Cottus sp.* complex for example, Nolte and Sheets [26] found a specific hybrid shape that was intermediate along the axes separating their parental groups, but that also displayed additional differentiation. In the *Chondrostoma* species complex, convergence of body shape and coefficient condition between the two species have been described for this model despite heterogeneous genetic patterns (i.e. different hybrid combinations and parental individuals). Corse *et al.*[19] reported that the mouths of the F1 hybrids display an extreme phenotype resulting from the lower lip widening slower than in the two parent species. Phenotypic diversity of F1 specimens is of particular interest, because the phenotypes of these individuals in some cases exceed the range of phenotypes in the corresponding parental lineages demonstrating transgressive segregation for the first time in backcrossed individuals [27,28]. This new character range constitutes a source of evolutionary novelties (i.e. transgressive segregation) on which selection could act. Thus, hybridization may contribute to evolutionary novelties in animal through the emergence of novel phenotypes due to transgressive segregation [3,26].

It would be valuable to be able to identify adaptive peaks or sets of pathways that spread in the adaptive landscape, and to describe the interactions between new genomic architectures and observed phenotypes in hybrid zones. To decipher the complex evolutionary history linking genotype and phenotypes in hybrid zones, two major conditions need to be respected. First, the genetic diversity of source populations has to be estimated as this is an important factor of morphological variation among admixed populations [25]. Second, it is important to model phenotypic variability among admixed individuals; such variability can change significantly within a few generations in a particularly dynamic process [29]. The change in variability depends on the distribution of admixed individuals in the hybrid zone, the environmental parameters, time and duration of hybridization, and the level of gene flow [18]. It would therefore be of interest to evaluate transgressive segregation in natural systems including in particular a recent hybrid zone. Indeed, an ancestral hybrid zone or a lineage of hybrid origin may be subject to secondary evolutionary processes that reshape the hybrid morphology and may have hidden the transgressive traits.

The *Chondrostoma* species complex appears to be a useful model for investigating how admixture between two divergent lineages (*Chondrostoma* species and another species belonging to a different genus) shapes morphological variation. *Chondrostoma* hybrid zones have been previously described in a fragmented habitat (the Durance River), assuming that dams favour hybridization between *Parachondrostoma toxostoma* and *Chondrostoma nasus*[17]. With this model, we can identify source populations for each species, recent hybrid zones and it is possible to genotype large numbers of new hybrid specimens from this habitat.

The aim of this study was to describe genetic-phenotypic interactions and to assess the transgressive traits in body shape and thereby to decipher the evolutionary dynamic of this “natural” Chondrostoma hybrid zone. Recently, intermediate individuals were found in the Ardèche River (a non-fragmented river) raising questions about the capacity of such zone to generate large numbers (and diversity) of hybrids. We report a study of 41 microsatellites, partial *cytochrome b* gene sequences and body shape morphology in 970 specimens from (i) a non-fragmented hybrid zone and (ii) source populations including allopatry and parapatry for both species (*Parachondrostoma toxostoma* and *Chondrostoma nasus*). To study interactions between morphological changes and genetic architecture in hybrid zones, we combined clustering methods [22] and the genomic cline approach [24] to explain genotype as a function of hybrid index and test potential effects of selection. The findings were then used to describe body shape transformation (identified by a morphometric analysis) along the h-index gradient. Finally, we interpret morphological variations linked to hybridization, taking into account ontogenetic effects on shape.

## Results

### Hardy-Weinberg equilibrium, null allele frequencies and discriminant power of microsatellites

Most microsatellite markers (allopatry and parapatry) were in Hardy-Weinberg equilibrium in the reference populations of *C. nasus* (88-95%) and *P. toxostoma* (76-83%). However, two populations in the Ardèche, more loci showed a deficit or excess of heterozygosity (Rosières: 61%; Saint-Just: 85.4%; See Additional file 1: “Observed and expected heterozygosity for each marker by population”). This was due to the presence of both species in samples from the Ardèche. However, the Labeaume population presented 35 loci in Hardy-Weinberg equilibrium (85%) indicating panmixia between specimens of *C. nasus*, *P. toxostoma* and hybrids.

Considering the loci which have null allele frequency higher than 0.05, 16.5% was found in reference populations (See Additional file 2: “Estimates of the frequency of null alleles by population”). In *P. toxostoma*, the distribution of such loci ranged from 7.31% to 9.76% for and from 19.51% to 34.15% for *C. nasus*. In hybrid zone, 14.63% of such loci were found in Labeaume population (the only panmictic population).

**Figure 1:**
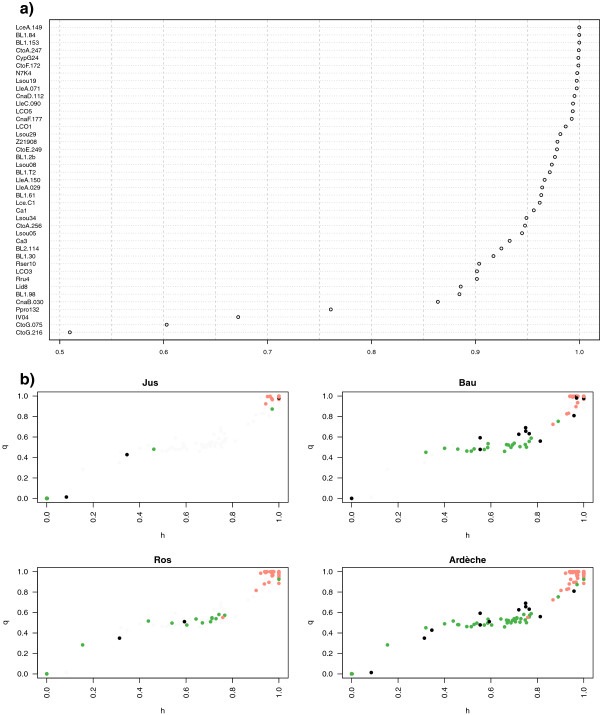
**Power of the 41 microsatellites loci. a**) Discrimination score (abscissa) for each of the 41 loci (ordinate). The discrimination score considered here is the area under the ROC curve which plots the sensitivity (ie probability to detect true positif) in function of the inverse of the specificity (probability to detect true negatif), after performing a logistic model explaining species status by allele composition. Scores of 0.5 indicate a monomorphic locus (a single, same allele in both species) or a polymorphic locus (the same several alleles in both species); scores of 1 indicate to a locus at which the two species studied do not share any allele. **b**) For the three Ardèche stations separately and together: q score (from Structure analysis) as a function of the h-index (from Introgress analysis) with 0 corresponding to *C. nasus* and 1 to *P. toxostoma*. In green, specimens with *C. nasus* mitochondrial DNA, in red specimens with a *P. toxostoma* mitochondrial DNA, in black mitochondrial DNA typing not available.

Most of the microsatellites are highly discriminant (Figure 1a). Comparing the results of the two methods (Structure and Introgress), we showed congruence in assignment of individuals (Figure 1b, see Additional file 3: “Assignment of individuals with Introgress and estimation of h-index”).

### Pattern of the Ardèche hybrid zone versus reference populations

**Figure 2:**
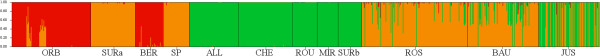
**Admixture between populations of *P. toxostoma *and *C. nasus *(K = 3).** Red and orange: *P. toxostoma*; green: *C. nasus*.

The LnP(D) and ΔK analyses of the data for the 41 microsatellites to determine the K parameter showed a significant number of groups with K = 3 (Figure 2, See Additionnal File 4b: “Admixture with Structure software between populations of *P. toxostoma* and *C. nasus.*”). This led us to differentiate *P. toxostoma* reference populations into two groups: Rhône basin populations (Suran River, Serre-Ponçon Lake), and coastal river populations (Orbieu River, Berre River). However, there was no differentiation of *C. nasus* populations.

**Figure 3:**
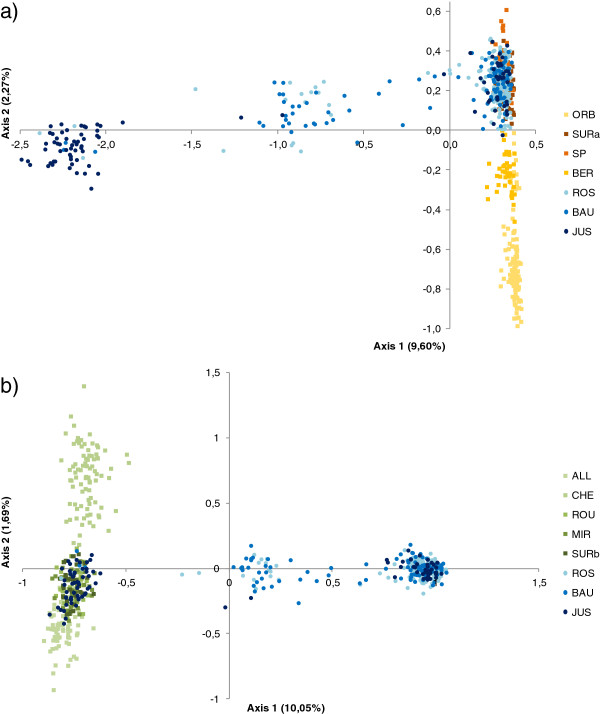
**Multiple Correspondance Analysis (MCA) for the 41 microsatellites. a**) MCA of *P. toxostoma* reference populations and Ardèche populations. **b**) MCA of *C. nasus* reference populations and Ardèche populations.

Bayesian analyses were confirmed by multiple correspondence analysis (MCA), and indicated that *P. toxostoma* individuals in the Ardèche were most closely related to Rhône populations of *P. toxostoma* (Figure 3). *C. nasus* individuals in the Ardèche were more closely related to *C. nasus* populations in the Rhône and Allier rivers than that in Chée. This last result appeared in Bayesian analyses with K = 5 but was not significant (See Additional file 4: “Admixture with Structure software between populations of *P. toxostoma* and *C. nasus*”).

### Characterization of admixture in the Ardèche hybrid zone

**Figure 4:**
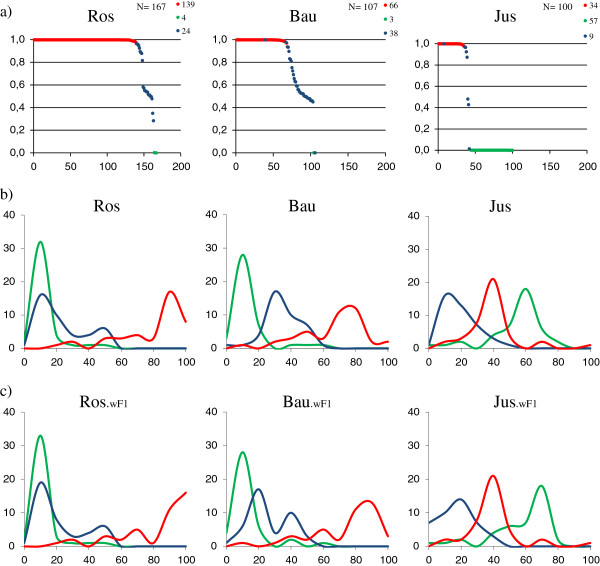
**Introgression pattern in populations of the Ardèche. a**) Distribution of individuals in the Ardèche hybrid zone correlating the percentage of ancestral genome (q score of Structure) defined with microsatellite data and mitochondrial DNA (with *P. toxostoma* in red, *C. nasus* in green, and hybrids in blue, see text). **b**) and **c**) Distribution of the 41 loci according to the percentage of homozygous and interspecific heterozygous specimens given by Introgress (*P. toxostoma* in red, *C. nasus* in green, and hybrids in blue) in Ardèche populations (**b**) and in Ardèche populations without F1 individuals (**c**). As an example, in the Rosières population, at more than thirty loci 10% of the specimens are *C. nasus* homozygous.

Based on reference specimens, the ranges of h-values for *C. nasus* (resp. *P. toxostoma*) were [0.00, 0.087] (resp. [0.940, 1.00]), see material and methods). The “pure” specimens observed in Ardèche River ranged from 0.00 to 0.04 for *C. nasus* and from 0.946 to 1.00 for *P. toxostoma*, with a congruent maternal heritance of mitochondrial DNA (*C. nasus* mitochondrial DNA between 0.00 and 0.04; *P. toxostoma* mitochondrial DNA between 0.946 and 1.00). Consequently, hybrids correspond to (i) individuals that are ranged from 0.04 to 0.946 regardless mitochondrial DNA and (ii) to individuals for which the microsatellites and mitochondrial DNA were not congruent (Figure 4).

The percentage of hybrid individuals was higher in the upstream part of the river (11.20% in Rosières and 31.50% in Labeaume) than in the downstream part (4.90% in Saint-Just) where the two species overlapped. The proportion of hybrid specimens with *C. nasus* mitochondrial DNA was 76.47% (13/17) at Rosières, 87.50% (21/24) at Labeaume, and 75.00% (3/4) at Saint-Just. Introgression of *P. toxostoma* mitochondrial DNA into individuals with a pure *C. nasus* nuclear genome (based on the 41 microsatellite loci) was 0.00% (0/4) at Rosières, 0.00% (0/3) at Labeaume and 2.17% (1/46) at Saint-Just. Introgression of *C. nasus* mitochondrial DNA into the individuals with a pure *P. toxostoma* nuclear genome was 0.69% (1/144) at Rosières, 0.00% (0/63) at Labeaume and 6.00% (2/33) at Saint-Just (Figure 4).

The hybridization in Ardèche was asymmetrical, with many individuals being the result of backcrosses with *P. toxostoma*. Patterns of hybridization were particularly different between the three stations on the Ardèche: a sigmoid line with numerous pure *P. toxostoma* individuals and some hybrids in Rosières; *P. toxostoma* individuals and more hybrids in Labeaume; then pure individuals of *C. nasus* and *P. toxostoma* with fewer hybrids in Saint-Just (Figure 4).

Most hybrid specimens presented a h-index closer to “pure” *P. toxostoma* (Figure 4)*.* The distribution of the 41 microsatellite markers in hybrid specimens without F1 individuals displayed a bimodal distribution between loci within genome (especially in Rosières and Labeaume populations) indicating heterogeneity in the genomic porosity (Figure 4b, c).

### Genomic architecture and admixture: the genome-environment interaction

Most of the loci studied have evolved under the neutral model: 51.22% to 56.10% (respectively with F1 individuals and without F1 individuals) for Rosières, 56.10% to 58.54% Labeaume and 85.37% to 78.05% for Saint-Just (Table 1, see Additional file 5: “Determination of marker genomic cline for each population of Ardèche and populations without F1 individuals”). The downstream part of the river (Saint-Just) appears to be the tail of the hybrid zone. However, for the upstream part of the river, different selective forces apply to hybrid genomes in different localities. The most important selective force identified (respectively with F1 individuals and without F1 individuals) differed between the two stations: positive selection of invasive homozygotes *C. nasus* was observed for nine to seven loci (21.95% to 17.07%) in Rosières and four to six loci (9.76% to 14.63%) in Labeaume. Overdominance selection of interspecific heterozygotes (without F1) was detected for five loci (12.20%) in Rosières and Labeaume for which three are common. The overdominant selection corresponded to the second most important selective force in Rosières and Labeaume (Table 1, See Additional file 5: “Determination of marker genomic cline for each population of Ardèche and populations without F1 individuals ”).

**Table 1:** Number and percentage of loci corresponding to a selection model by populations in Ardeche and populations without F1

**Selection model**	**Ros**		**Ros.wF1**		**Bau**		**Bau.wF1**		**Jus**		**Jus.wF1**	
	**N**	**%**	**N**	**%**	**N**	**%**	**N**	**%**	**N**	**%**	**N**	**%**
neutral	21	51,22	23	56,10	24	58,54	28	68,29	35	85,37	32	78,05
positive selection	7	17,07	5	12,20	2	4,88	4	9,76	3	7,32	2	4,88
overdominance	4	9,76	4	9,76	4	9,76	2	4,88	0	0,00	0	0,00
overdominance/positive selection	0	0,00	0	0,00	1	2,44	0	0,00	0	0,00	0	0,00
overdominance/negative selection	1	2,44	0	0,00	2	4,88	1	2,44	0	0,00	0	0,00
overdominance/epistasis	1	2,44	1	2,44	0	0,00	2	4,88	0	0,00	0	0,00
underdominance	1	2,44	1	2,44	3	7,32	0	0,00	0	0,00	2	4,88
underdominance/positive selection	2	4,88	2	4,88	1	2,44	2	4,88	0	0,00	0	0,00
increase admixture	2	4,88	2	4,88	3	7,32	1	2,44	1	2,44	0	0,00
epistasis	1	2,44	1	2,44	0	0,00	0	0,00	1	2,44	0	0,00
monomorphic	1	2,44	1	2,44	1	2,44	1	2,44	1	2,44	1	2,44
undefined selection	0	0,00	1	2,44	0	0,00	0	0,00	0	0,00	4	9,76

(positive selection is in the invasive homozygote *C. nasus* direction). Ros, Bau, Jus corresponded to Rosières, Labeaume, Saint-Just stations.

**Figure 5:**
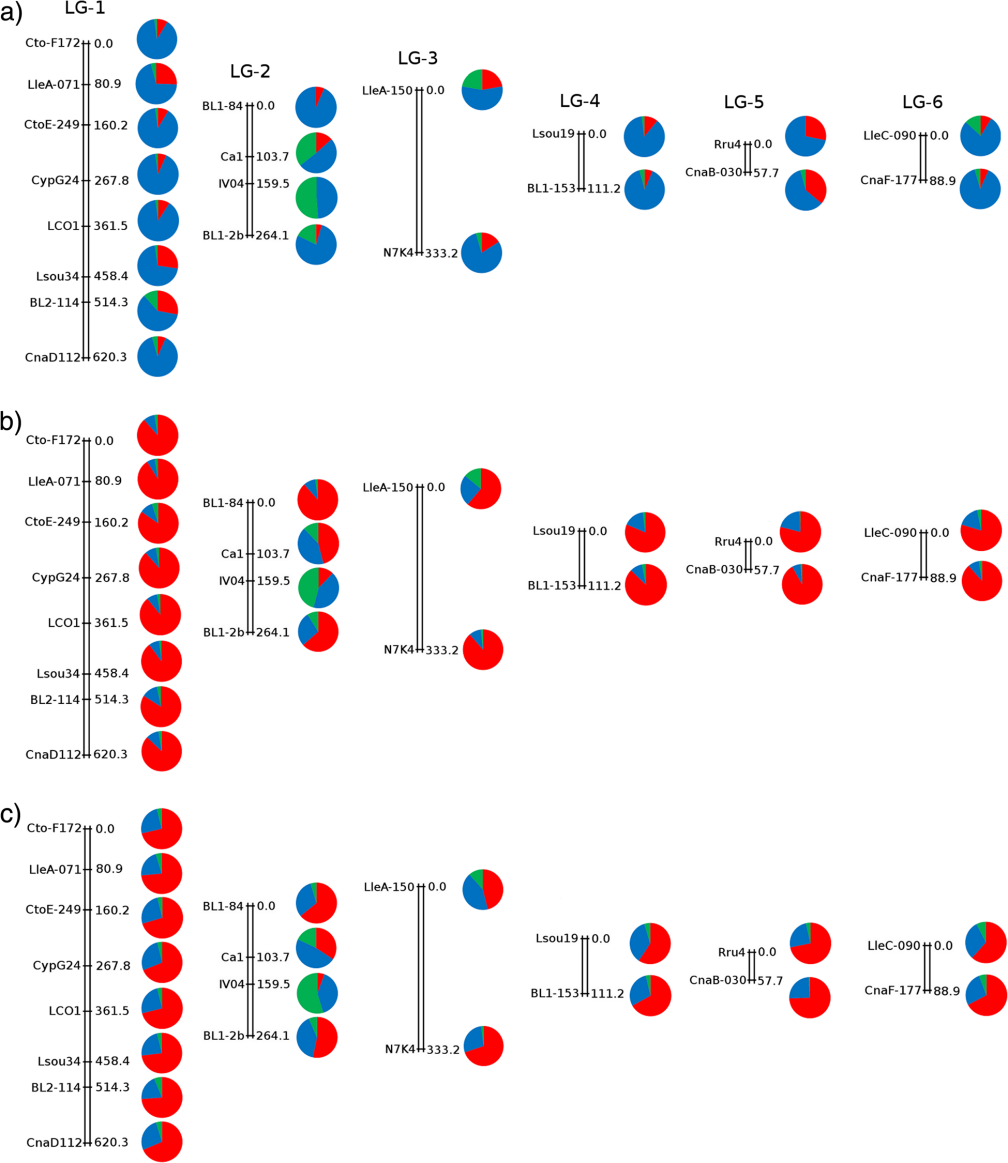
**Linkage map of 20 *loci *showing heterogeneity in allelic distribution of specimens.***P. toxostoma* in red, *C. nasus* in green, and hybrids in blue: **a**) for the 47 hybrid specimens from the Rosières and Labeaume populations; **b**) for all individuals from the Rosières population; **c**) for all individuals from the Labeaume population.

Based on hybrid specimens, six linkage groups (LG) were detected and permitted the association of 20 of the 41 loci (Figure 5) using the range 1.5-10; three linkage groups were identified using the range 2.0-5.0 (LG-1, LG-2 and LG-6). We describe the linkage groups based on the most complete pattern (i.e. six linkage groups) but we base our conclusions only on LG-1, LG-2 and LG-6. LG-1 includes eight loci, LG-2 four loci and the four other LGs contain two loci each. These LGs have not evolved at the same rate: LG-5 is characterized by hybrid and *P. toxostoma* genomes, LG-3 and LG-2 by the presence of the *C. nasus* genome, and LG-1 and LG-6 by a hybrid genome (interspecific heterozygote). Indeed, four overdominant loci (from a total of five) were found in the first part of LG-1.

Eighteen of the loci have flanking regions homologous to those in *Danio rerio*; eight of these 18 are present in the linkage groups (Figure 5). CypG24, BL2-114 and CnaD112 belong to the LG-1, but map to different chromosomes in *D. rerio* (Dr7, Dr17 and Dr1, respectively). By contrast, BL1-84 and Ca1 belong to LG-2 and are both present on Dr5; LleC-090 and CnaF-177 defining LG-6 are linked on Dr8, and CnaB-030 belonging to the LG-5 is present on Dr 1 (Figure 5).

### Multiple trajectories in hybrid body shape

**Figure 6:**
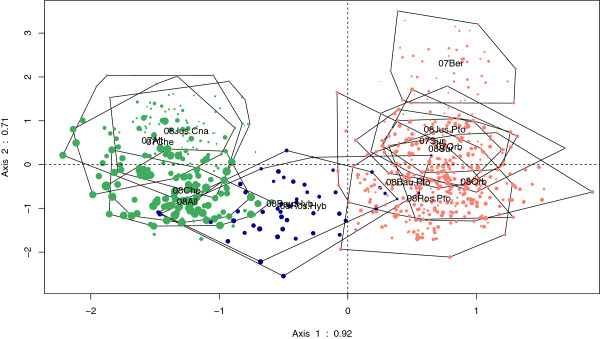
**Discriminant analysis of body shape.***P. toxostoma* in red, *C. nasus* in green and hybrids in blue, losanges correspond to supplementary specimens (see text).

The PCA of body shape in the reference populations indicated that the main component of variability was related to species. Body shape variation between groups defined by species and hybrids by station and year was described by DA analysis (Figure 6). The two first axes were associated with a substantial morphological differentiation (λ = 0.92 and λ = 0.71). The first axis corresponded to a species differentiation while the second axis ordered the populations according to specimen size (from small specimens to large specimens) whatever the species considered. Thus, this axis appears to be related to an ontogenetic modification. This result was confirmed by comparison of specimen size between populations/groups (See Additional file 6: “Box-plot of size for each reference populations and Ardèche populations by year”).

The pure specimens from Ardèche correspond to the ranges of the two reference species on the two DA axes. The Ardèche hybrids displayed new body shape morphologies that occupied the range between the two species on the first axis. Co-inertia analysis indicated a significant linear relationship between shape and microsatellites (See Additional file 7: “Co-inertia analysis”). Indeed, genomic dilution (h-index) and body shape deformation (from *C. nasus* to *P. toxostoma*) were correlated (r = 0.64 in Rosières to r = 0.88 in Saint-Just, P < 10e-6, permutational test). However, we did not identify any locus or group of loci related to a particular shape modification.

**Figure 7:**
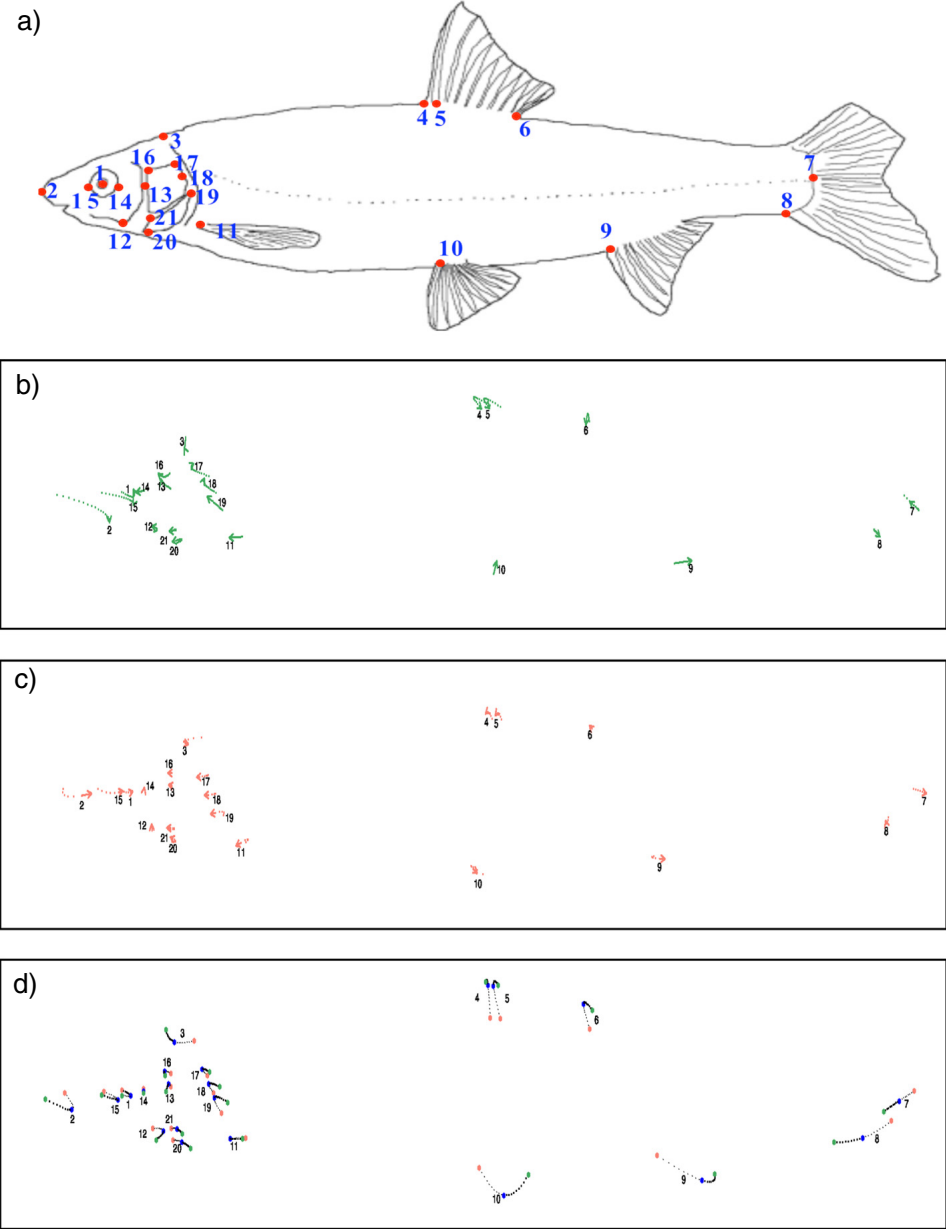
**Shape deformation for pure specimens and hybrids. a**) The 21 landmarks chosen for morphologic analysis **b**) Mean ontogenetic deformation for *C. nasus,* with progressing age, in reference conditions. **c**) Mean ontogenetic deformation for *P. toxostoma,* with age, in reference conditions. **d**) Deformation between *P. toxostoma* (h = 1, Ardèche specimens) and *C. nasus* (Chée and Allier stations in 2008, see text). In blue, form of the intermediate hybrid (h = 0.5).

The ontogenetic deformation was heterochronic [30]: the different landmarks evolved at different rates in the two species (Figure 7). Note that the *C. nasus* specimens from Saint-Just were small (and young). This could have led to a misleading description of shape hybridization. To avoid this potential problem due to ontogenetic effect, we used specimens from Chée and Allier (allopatric populations) from the year 2008 rather than the Saint-Just *C. nasus* population in the analysis. We then studied the morphological pathway between species: surprisingly, the observed mean hybrid (specimen displaying a h-index equal to 0.5) differed from the hypothetical hybrid (h = 0.5), indicating that body shape dilution was not linear (Figure 7). These differences were not only due to the intensity of vector modification (depending on the species tendency) but also on the direction of the vector. We demonstrated that hybrid body shape is a complex phenomenon that combines heterochronic processes between the two species in a non-linear way (Figure 7). Hybrid specimens (based on h-index) present a mosaic of characters: many are those of *C. nasus* (e.g. landmark 9 corresponding to the anal fin insertion), or of *P. toxostoma* (e.g. landmark 2 corresponding to the extremity of the mouth), and some are intermediate morphs (e.g. landmark 7 corresponding to the last scale in the lateral line), and transgressive segregation morphs (e.g. landmark 11 corresponding to the pectoral fin insertion), or similar to the two parental species (e.g. landmark 14 corresponding to the right side of the eye positioned on the line between the landmarks 13 and 2). Thus, hybrids displayed a shorter snout pointing down, a smaller eyes (leading to a shorter head) and greater body height than either of the two parental species.

## Discussion

### Plasticity of genomic architecture in hybrid zone

The Ardèche hybrid zone presents neutral and selective forces, which are not homogeneous in the river. Indeed, our results suggest that the patterns of introgression at the downstream station (Saint-Just) were consistent with a neutral diffusion model involving genome-wide admixture whereas different selective forces affect rates of introgression for 50% of the genome at two sites (Rosières and Labeaume, the upstream part of the Ardèche basin). The most important selective force at Rosières appeared to be a positive selection of invasive *C. nasus* homozygotes (17.07%) indicating that hybridization may increase the fitness of admixed individuals. However, the percentage was substantially lower than the 30% found in the sculpin hybrid [16]. The most important selective force operating in the upstream parts of the river (Rosières and Labeaume) appeared to be an overdominant fitness of heterozygotes (12.20%) evidence of genomic compatibility between the two species for the loci considered. This was especially true for the Labeaume population which was 31.50% hybrids, and almost all the loci were in Hardy-Weinberg equilibrium. This population is an example of panmixia between specimens from *C. nasus*, *P. toxostoma* and hybrids, despite striking genetic differentiation between the two species. Finally, the underdominance of interspecific heterozygotes observed at the Rosières and Labeaume sites (4.88%-7.32%) may reflect genomic isolation (incompatibility) between the two species and/or lower fitness of heterozygotes in this environment. As indicated by Nolte *et al.*[16], an underdominance signal may be an artefact due to a high frequency of null alleles in hybrids. However, in our study the high frequency of null alleles can be an artefact only at the Lid8 locus (See Additional file 2).

The map distances between several of the loci studied are relatively large, and the map order of some of the markers can only be predicted from the *Danio rerio* genome. Nevertheless, the loci implicated in overdominant fitness of heterozygotes can be identified (see Figure 5). Our genomic mapping of specimens from the Ardèche hybrid zone revealed that the six loci presenting overdominance are clustered into two LG: four are members of LG-1 (which includes a total of eight loci) and two are in LG-2 (LG-2 contains four loci, Figure 5). These findings may indicate a chromosomal region at which heterozygosity confers an advantage, or particular regions of the chromosome displaying a hitchhiking effect. However the loci involved in other selective forces (positive selection or underdominance) could not be mapped. Additional microsatellite markers need to be developed and mapped to help decipher the contribution of the various evolutionary forces to the hybrid genomic architectures observed.

The divergence between the two species is almost 7% on *cytochrome b* of mitochondrial DNA [31], and a large number of loci have high discriminant scores (25 loci with a discriminant score >0.95, Figure 1a). Consequently, a large number of interspecific heterozygotes at numerous loci would not be expected in hybrid generations beyond the F1 [32]. However, in our study were detected in Labeaume population 31.50% of hybrid specimens and 12.20% of the loci presented an overdominant fitness of heterozygotes. A similar result was found in the hybrid zone in Populus [33]. The authors suggested that epistatic interactions between loci best explain the heterozygote advantage of recombinant hybrids. Both species may have co-adapted gene complexes and, possibly, only hybrids with two complete sets display high fitness. In the *Cottus* model, a set of transgressively over-expressed genes in the invasive hybrid lineage is a non-random set that is functionally linked; also, a large subset of these genes appeared to be plastic [34]. These two previous studies and our findings suggest therefore that the overdominant fitness of heterozygotes may be a major source of evolutionary novelties.

### Hybrid zone as a source of phenotypic novelties

Phenotype-genotype associations are a major topic of research in evolutionary studies [12,35]. Pigliucci [36] states that an understanding of the sources of variation underlying the evolution of novel complex phenotypes is one of the key challenges in biology. Mathematical models can be used to test for associations between genetic composition and complex sets of correlated traits [37]. However it is not straightforward to take ontogenetic parameters into account, especially in studies involving hybrid zones. Here, we describe an analytical framework in which the complex interactions between morphological changes and genetic architecture in hybrid zones are taken into account.

Hybridization between *P. toxostoma* and *C. nasus* produces a range of hybrid specimens such that there are two different asymmetrical distributions. In our study system, the upstream part of the river presented specimens introgressed by *P. toxostoma* while the downstream part displayed specimens introgressed by *C. nasus*. This new genomic landscape, constituted by recombinant specimens (the hybrids), is the scene of complex body shape evolution. Indeed, body shape does not constitute a monolithic block but a heterogeneous structure presenting a complex pattern of correlated traits. Ontogenetic trajectories represent emergent processes resulting from interaction between an individual’s genotype and its environment. The new genomic architectures of Ardèche hybrids interact with environmental cues and thereby generate new phenotypes via the ontogenetic trajectories followed by each individual. We found that the morphology of hybrids between *C. nasus* and *P. toxostoma* is not a linear transition between the two parental species; it is a mix of characters within the range bounded by the two parental species but also of characters outside this range (i.e. transgressive segregation). The landmark trajectories could be *C. nasus* dominant, *P. toxostoma* dominant, *C. nasus*/*P. toxostoma* co-dominant or different from those of both species making each hybrid an evolutionary novelty. We provide clear evidence that Ardèche hybrids follow complex ontogenetic trajectories due to a heterochrony and heterotopy (as defined by Zelditch and Fink [38]) of the various morphological traits.

### Hybrid zone: general pattern or particular cases?

Hybridization is a diverse process that can have many forms. Typical cline models are found in most hybrid zones, and patterns in which both parental species are found along the entire range (mosaic pattern) are more unusual; diverse examples have been described and differ between species and models [39]. Nevertheless, the highly divergent patterns of hybridization we report between two species are uncommon. The Ardèche hybrid zone displayed a clearly sigmoid cline, contrasting with the mosaic hybrid zone in the Durance River [17]. The presence of hybrids at the four Durance collecting sites separated by dams indicated that human activity has disrupted the ecology of both species, which share an overlapping ecological niche. There were more hybrids in the upstream and middle parts of the river (33.5% in the less fragmented part) and specimens belonging to the invasive *C. nasus* were abundant at the two most separated stations: 32% for the upstream station and 62% for the downstream station (versus 2.0-5.5% for the intermediate stations). In Ardèche, the hybrids were most abundant in the upstream part of the river (21.35% of the Rosières and Labeaume populations) although specimens of the invasive species (*C. nasus*) were rare (2.86%). The *C. nasus* nuclear genome was rare in the hybrid specimens whereas more than 81.99% of the hybrid specimens carried *C. nasus* mitochondrial DNA. This indicates that most hybridization involved female *C. nasus* reproducing with male *P. toxostoma*.

Hybridization of Chondrostoma complex species does not depend on the presence of dams and indeed occurs whatever the habitat (both highly fragmented habitats and natural habitats). However, patterns of hybridization differ very substantially between two habitats, implying that introgressive processes follow different pathways in different environments.

We confirmed that the inheritance from the rarer species, as described by Costedoat *et al.*[17] in the Durance River, is also present in the Ardèche: this feature therefore seems to be a characteristic of hybridization between *C. nasus* and *P. toxostoma* that is conserved, independent of the environment.

## Conclusion

Species ecology makes a major contribution to the characteristics of hybrid zones. In the system we studied, the invasive species (*C. nasus*) lives mostly in the downstream part of the Rhône River and spawns in tributaries (Suran River, Ardèche River as described by Nelva [40]). The spawning period is sufficient to produce fertile hybrids with the endemic species *P. toxostoma*. Hybridization between these two species is not driven by dams on the Ardèche basin, but dams do contribute to the observed pattern of hybridization in the Durance River. The cause of hybridization is unclear, particularly as there is a parapatric zone in the Rhône basin in which no hybrid (or introgressed genome) was found in either *C. nasus* or *P. toxostoma* populations. The consequences of ecological factors on the number and diversity of hybrids between divergent lineages should be studied. The Para/Chondrostoma species complex is a useful model for investigating the ecological factors responsible for these recent hybrid zones (created no more than 150 years ago) and their persistence. Finally, it would be interesting to decipher the evolution of genomic compatibility/incompatibility (endogeneous selection) between the two species and their hybrids, both in different hybrid zones and by more detailed analysis at the genomic scale.

## Materials and methods

### Sampling and molecular data

**Figure 8:**
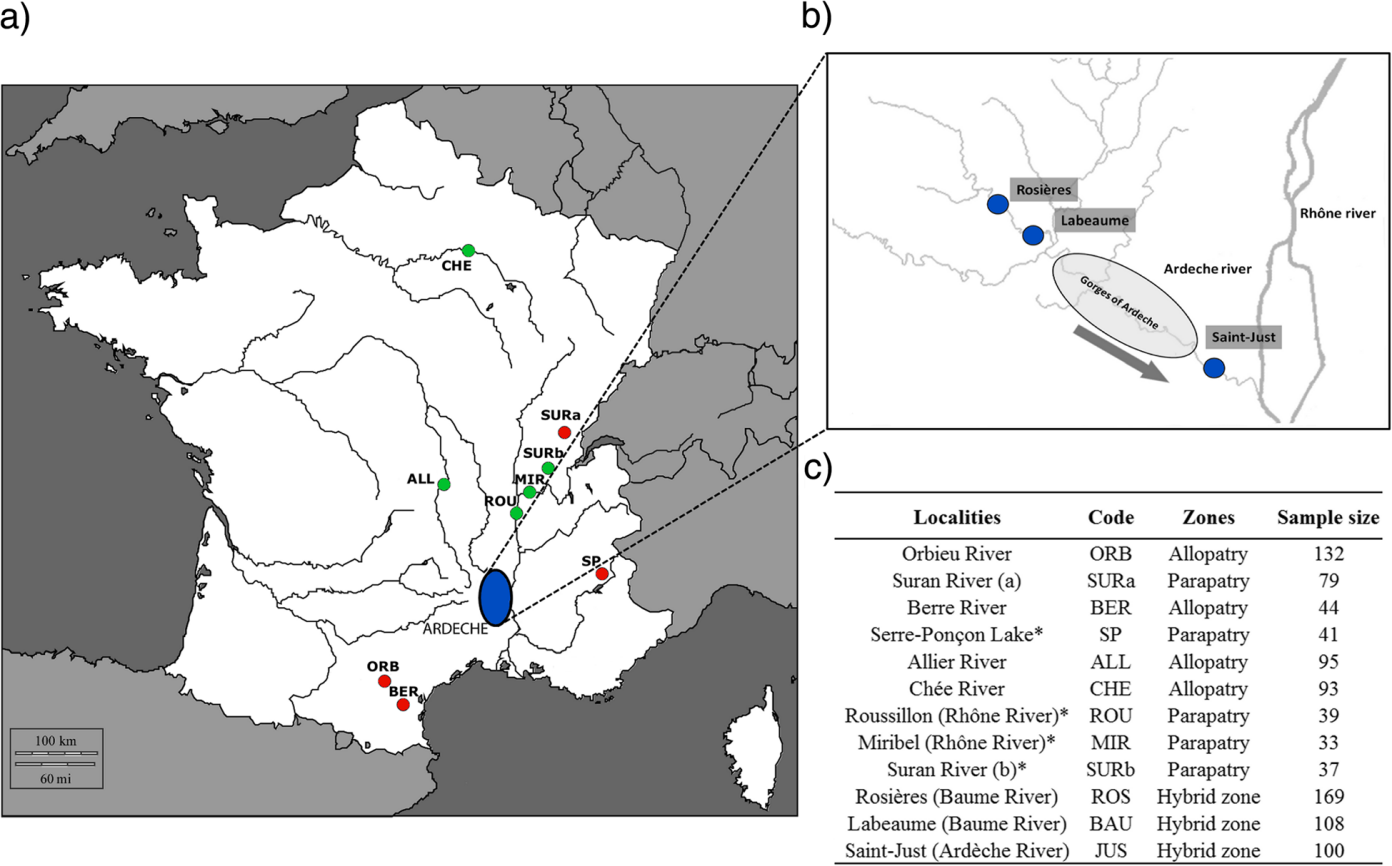
**Geographic distribution range. a**) Sampling stations in allopatric and parapatric zones for *Parachondrostoma toxostoma* (red circles : ORB, SURa, BER, SP); *Chondrostoma nasus* (green circles : ALL, CHE, ROU, MIR, SURb). **b**) Sympatric populations in the Ardèche (blue circles : ROS, BAU, JUS). c) Locality names and sample sizes for analysis of body shape, microsatellites and mitochondrial DNA. *Microsatellites and mitochondrial DNA only.

A total of 970 individuals were sampled from 12 localities in France over four years. Both parental Chondrostoma lineages were collected in allopatric and parapatric zones (2007 to 2010; 593 individuals); these allopatric and parapatric zones are referred to as “reference populations”. We also sampled a non-fragmented hybrid zone, the Ardèche basin (377 individuals), at three localities in 2008. The dowstream station (Saint-Just, Ardèche) is close to the junction with the Rhône River, the upstream station (Rosières, Baume) is the limit of the distribution of the endemic species (*P. toxostoma*) and the third station is 7 km downstream (Labeaume, Baume) from the upstream station. The two stations (Labeaume and Rosieres) are separated by a dry zone in the river without surface water during the warmest periods. The details of the populations and sample sizes are presented in Figure 8.

### Genetic data collection

Total genomic DNA was extracted from a piece of caudal fin, and a 500-bp fragment of the *cytochrome b* mitochondrial DNA gene was sequenced using standard polymerase reaction (PCR) procedures, as described in Costedoat *et al.*[31]. Seqscape 2.5 software (Applied Biosystem®) was used to align and correct sequences.

Forty-one microsatellite loci were amplified using five multiplex PCR kits as described in Dubut *et al.*[41]; the flanking regions of 19 of these loci in leuciscinae species are homologous to those in *Danio rerio*. Allelic designation was standardised using an allelic ladder to calibrate the various data obtained with samples collected in different years. The procedure is described in LaHood *et al.*[42]. GeneMapper 3.7 software was used for correction and genotyping [43].

### Genetic data analysis

The mitochondrial *cytochrome b* gene sequence was used to identify the maternal inheritance of hybrid individuals.

Hardy-Weinberg equilibrium within populations was estimated by comparing observed and expected heterozygosity for each locus with Arlequin 3.1 [44]. Then, to analyse the cause of departure from Hardy-Weinberg equilibrium, we used ML-NullFreq to estimate for each locus the frequency of null alleles in populations [45]. This generates estimates based on a maximum likelihood approach. Because no analytic expression of the estimates is available, the EM algorithm [46] was used to estimate the null allele frequency.

Genetix 4.05.2 was used for a multiple correspondence analysis (MCA) of 0/1/2 data (the genotypic state), as proposed by She *et al.*[47]. The complete data set (allopatry, parapatry and sympatry) for the two species and their hybrids was considered.

We establish a discriminant score for each of the 41 loci. The discrimination score considered here is the area under the ROC (Receiver Operating Characteristic) curve which plots the sensitivity (i.e. probability to detect true positif) in function of the inverse of the specificity (probability to detect true negatif), after performing a logistic model explaining species status by allele composition. Scores of 0.5 indicate a monomorphic locus (a single, same allele in both species) or a polymorphic locus (the same several alleles in both species); scores of 1 indicate a locus at which the two species studied do not share any allele.

### Individual admixture

#### Bayesian procedure

We used a Bayesian model-based clustering algorithm implemented in the software Structure 2.3 [22]. Individuals in the sample were assigned to K populations or a mix of populations if their genotypes indicate that they are admixed. We used the ‘admixture model’ and the “I-model” (independent allele frequencies). The burn-in length was set to 100,000 followed by 1,000,000 iterations within a Markov Chain Monte Carlo (MCMC) as recommended by the authors [22]. These analyses were carried out with K = 2 (the two species) up to 6 allowing for possible population structure within each species. We selected the K value for which the posterior probability of the data, LnP(D), was maximised, taking into account the ΔK correction of Evanno [48]. We also used the “q score” given by Structure to quantify genome ancestry through the populations.

### Introgress procedure

The hybrid index (h index) is simply defined as the proportion of alleles inherited from one of the two parental populations [49]. A maximum likelihood estimate of the h index has the advantage of taking uncertainty in inheritance into account, i.e. when *loci* do not exhibit fixed differences between the parental populations. The maximum-likelihood hybrid estimate of the h index for heterozygous genotypes is thus sensitive to parental allele frequencies [49].

The Introgress R package [50] was used to estimate the h index for each of the specimens and for the three stations on the Ardèche, separately.

To detect locus-specific selection, we used the procedure proposed by Gompert and Buerkle [50]. It involves developing a multinomial logistic model explaining the genotype (defined by three states: one heterozygous and two homozygous) according to the h index; significant departure from a neutral model is tested using a log likelihood ratio-test. Patterns of non-neutral loci are thereby described as underdominance, overdominance, epistasis, increase admixture or directional selection. Because F1 hybrids could induce a bias in the test, we use Newhybrids software [51] to detect F1 specimens and two separate analyses (with and without F1) were developped. We found 3 specimens in Rosières, 15 specimens in Labeaume, and 2 specimens in St-Just. The F1 specimens constituted 12.50% of the “hybrid population” in Rosières, 42.86% in Labeaume, and 28.57% in St-Just. It is clear that a majority of individual presenting a q-score between 0.45-0.55 are F1 (but not all). Populations considered without F1 in analysis are called “wF1”.

We used the false discovery rate (FDR) with the majoration proposed by Benjamini and Hochberg as $P_{i}\leq \frac{i}{m}x\;a$, with i = the rank of the initial *P*-value (from highest to the lowest), m = the number of loci, α = 0.025 [52].

### Linkage disequilibrium and linkage map

We used the JoinMap 4.1 software [53] to construct a genetic linkage map using 47 hybrid specimens (selected using both Structure and Introgress estimations) which were mostly the result of back-crosses. We use the RIx as population type codes to represent a population of recombinant inbred lines in the x-th generation (we use x = 2 for only back-crossed specimens and x = 25 for the number of generations since the *C. nasus* introduction). We used the independence LOD parameter with threshold ranges of 1.5 to 10.0 and of 2.0 to 5.0. This analysis was combined with linkage disequilibrium testing using Genepop software [54].

### Morphological analysis

We analysed the body shape of 743 specimens collected at five reference stations over two years (Orbieu, Suran and Berre rivers for *P. toxostoma*; Allier and Chée rivers for *C. nasus*) and at the three Ardèche stations. We used the landmark-based geometric morphometric method following the procedure described in Costedoat *et al.*[17] and Corse *et al.*[55]: 21 homologous landmarks were digitised with TpsDig software [56], and the generalised procruste analysis (GPA) procedure was used for a geometric morphometric analysis of their coordinates [57]. Body shape variability was described first by Principal Component Analysis (PCA) to identify the main factor of between-specimen variability. A linear discriminant analysis was performed with the same dataset, groups being defined for reference conditions by nine year x station combinations. In reference conditions, values of h different from 0 (or 1) reflect shared polymorphism between species. To estimate the h-value, we performed the introgress analysis on each of the reference populations. The resulting range of h-values for *Chondrostoma nasus* was [0, 0.087] and that for *Parachondrostoma chondrostoma* was [0.940, 1]. Hence, for the hybrid zone, we defined three classes according to the observed h-index 1) a specimen whose h is within [0, 0.087] will be considered as a *C. nasus,* hereafter encoded “Cna”; 2) a specimen whose h is in the range [0.940,1] will be considered as a *P. toxostoma* hereafter encoded “Pto”; 3) a specimen whose h-index does not fall within either of these two intervals will be considered as a hybrid, hereafter encoded “Hyb”. These three h-classes were crossed with stations to obtain the following groups: Saint-Just x “Cna”, Saint-Just x “Pto”, Labeaume x “Hyb”, Labeaume x “Pto”, Rosières x “Hyb”, Rosières x “Pto”. Three combinations were not taken into account because the samples were too small: Saint-Just x “Hyb”, Labeaume x “Cna”, and Rosières x = “Cna”. To interpret discriminant axes taking into account size effect, we initially studied size and age distributions by group. As form (resp. genotypes) corresponds to several variables (resp. microsatellites), the logic to study their covariation is to generalize univariate correlation. To do that, we consider co-inertia analysis [58]. The two following matrices restricted to the hybrid zone specimens (n = 363) were considered: X, the n (=363) x p (=42) shape variables (21 landmarks for abscissa and ordinate); and Y, the n (=363) x 42 (42 = 41 genotypes coded in three classes via the introgress procedure plus the h-index value). Co-inertia analysis builds a set of orthonormal pairs of axes (one for X and one for Y), with linear combinations of the variables and maximizing their covariance. The significance of the linear relationship between the two members of a pair was tested using a permutation test. We explored the possibly non-linear relationship between shape and h-index (hybrid-index) by modelling the relationship between each coordinate (resulting from the procruste superimposition) and h via height parametric additive models: polynomial models of degree 1 (i.e. simple linear model) to five, and piecewise cubic polynomial models with one and two interior knots. The Akaike information criterion [59] was used to select the best model. This model describes body shape transformation along the h-index gradient, taking into account possibly non-linearity as well as heterogeneous speed of shape modifications. To interpret morphological variations linked to hybridization, taking into account of ontogenetic effects on shape, we also performed a similar analysis replacing h by centroid-size separately for each of the two species in reference conditions.

## Competing interests

The authors declare that they have no competing interests.

## Authors’ contributions

AG CC EC JMO NP RC performed the field work. AG and NP conceived and designed the experiments. CC and EC acquired morphological data. MS acquired molecular data. NP conducted morphological analyses. MS and NP conducted molecular analyses. AG, MS and NP interpreted the data. AG wrote the first draft and CC, EC, MS and NP contributed to writing and revising the manuscript. All authors read and approved the final manuscript.

## Supplementary Material

Additional file 1 **Observed (Ho) and expected heterozygosity (He) for each marker for each population** (* *p-value* < 0.05, “-” means monomophic loci). Click here for file

Additional file 2 **Estimates of the frequency of null alleles for each population and marker.** With k visible alleles per locus (frequencies <0.01 in italic, >0.05 in bold; “-” indicates monomophic loci). Click here for file

Additional file 3 **Assignment of individuals withIntrogressand estimation of h-index.** a) for Rosières b) for Labeaume c) for Saint-Just. (green: h = 0 corresponding to *C. nasus* assignment, blue: h between 0 and 1 corresponding to hybrid assignment, and pink: h = 1 corresponding to *P. toxostoma* assignment). Click here for file

Additional file 4 **Admixture withStructuresoftware between populations of*P. toxostoma*and*C. nasus.*** a) Graphs of admixtures of populations from K = 2 to K = 6. b) Posterior probability (LnP(d)) and ΔK as a functions of K group. Click here for file

Additional file 5 **Determination of marker genomic cline for each population of the Ardèche and populations without F1 individuals.** (DS+: positive directional selection of invasive *C. nasus* homozygotes; DS-: negative directional selection of *C. nasus* homozygotes; OD: overdominance for interspecific heterozygotes; UD: underdominance for interspecific heterozygotes; EP: epistasis; IA: increase admixture; M: monomorph; “-” indicates neutral loci). Click here for file

Additional file 6 **Box-plot of size for each reference population and Ardèche population for each year.** (in green *C. nasus*, in red *P. toxostoma* and in blue hybrids). These individuals are those considered in the DA. Click here for file

Additional file 7 **Co-inertia analysis.** Plot of first co-inertia analysis axis for body shape as a function of first co-inertia analysis for microsatellites (green: h = 0, yellow: 0 < h < 0.3, blue: h between 0.3 and 0.8, pink: 0.8 < h < 1, red: h = 1). Click here for file
