# Supplementary material for: Non-homogeneous combination of two porous genomes induces complex body shape trajectories in cyprinid hybrids
Source: Front Zool. 2013 May 1;10:22. doi: 10.1186/1742-9994-10-22 (PMC3664599; doi:10.1186/1742-9994-10-22)
Supplement: Additional file 1 — Observed (Ho) and expected heterozygosity (He) for each marker for each population (* p-value < 0.05, “-” means monomophic loci). [file 1742-9994-10-22-S1.pdf]

	ORB (124)		SURa (70)		BER (44)		SP (41)		ALL (77)		CHE (85)		ROU (39)		MIR (33)		SURb (37)		ROS (167)		BAU (107)		JUS (100)	
	Ho	He	Ho	He	Ho	He	Ho	He	Ho	He	Ho	He	Ho	He	Ho	He	Ho	He	Ho	He	Ho	He	Ho	He
BL1-153	-	-	-	-	-	-	-	-	0.260	0.265	0.271	0.252	0.282	0.373	0.424	0.403	0.324	0.444	0.114*	0.156	0.299	0.324	0.240*	0.618
BL1-2b	0.460	0.442	0.300	0.277	0.273	0.238	0.537	0.552	0.727	0.708	0.647	0.696	0.744	0.740	0.455*	0.752	0.784	0.767	0.578*	0.606	0.604	0.601	0.6100*	0.811
BL1-30	0.532	0.592	0.771	0.761	0.455	0.400	0.683	0.693	0.701*	0.726	0.882	0.843	0.872	0.775	0.727*	0.728	0.838	0.753	0.671	0.711	0.632*	0.687	0.740	0.757
BL1-84	-	-	0.014	0.014	-	-	-	-	0.701	0.702	0.812	0.775	0.846	0.774	0.848	0.779	0.730	0.796	0.121*	0.144	0.318	0.323	0.530*	0.776
Lce-C1	0.290	0.277	0.714	0.681	0.409	0.476	0.439	0.429	0.533*	0.603	0.600*	0.630	0.615	0.631	0.576	0.634	0.456*	0.628	0.629	0.622	0.679	0.713	0.580*	0.793
LleA-029	0.234	0.218	0.676	0.722	0.636	0.754	0.925	0.820	0.662*	0.783	0.753*	0.886	0.579*	0.842	0.697*	0.853	0.722*	0.867	0.849	0.845	0.811	0.853	0.830*	0.894
LleA-071	-	-	-	-	-	-	0.268	0.333	0.014*	0.213	0.131*	0.235	0.103*	0.186	0.091	0.142	0.088*	0.192	0.072*	0.125	0.224*	0.282	0.100*	0.541
LleC-090	0.653	0.679	0.857	0.871	0.795	0.836	0.927	0.901	0.610	0.632	0.565	0.550	0.538	0.520	0.424	0.438	0.541	0.546	0.904	0.930	0.888	0.930	0.740*	0.833
Lsou19	0.137	0.169	0.243	0.222	0.455	0.398	0.195	0.200	0.779	0.763	0.859*	0.815	0.769	0.804	0.667	0.758	0.784*	0.833	0.404	0.408	0.542	0.520	0.610*	0.819
BL1-98	0.556	0.645	0.429	0.557	0.591	0.599	0.725	0.663	0.610	0.636	0.765	0.755	0.789	0.728	0.774	0.729	0.703	0.728	0.629	0.670	0.575	0.666	0.710	0.742
BL2-114	0.050	0.049	0.029	0.028	0.091	0.088	-	-	0.816	0.699	0.671*	0.742	0.743	0.725	0.563	0.605	0.556	0.602	0.168*	0.207	0.265*	0.328	0.480*	0.705
LceA-149	-	-	0.014	0.014	-	-	-	-	-	-	-	-	-	-	-	-	-	-	0.108*	0.156	0.280	0.332	0.040*	0.490
LleA-150	0.040	0.040	0.629*	0.779	0.682*	0.761	0.659	0.667	0.740	0.719	0.682*	0.862	0.795	0.823	0.758	0.781	0.730	0.842	0.844	0.871	0.819	0.904	0.850*	0.900
Lsou05	0.444	0.370	0.757	0.696	0.682	0.693	0.634	0.751	0.714	0.709	0.847	0.799	0.641	0.740	0.788	0.762	0.784	0.724	0.729	0.773	0.748	0.809	0.720	0.787
Lsou08	0.032	0.032	0.357	0.345	-	-	0.585	0.519	0.649	0.603	0.718	0.670	0.718	0.649	0.545	0.671	0.595	0.655	0.355	0.389	0.551	0.549	0.550*	0.731
Lsou29	0.742	0.748	0.914	0.901	0.727	0.788	0.976*	0.880	0.779	0.774	0.635*	0.791	0.564*	0.777	0.636*	0.815	0.730*	0.826	0.862*	0.913	0.913	0.921	0.800*	0.895
Lsou34	0.355	0.377	0.371	0.370	0.114	0.108	0.293	0.311	0.247*	0.335	0.471*	0.777	0.359*	0.622	0.394*	0.640	0.432*	0.638	0.608*	0.581	0.562	0.652	0.470*	0.788
Ppro132	-	-	-	-	-	-	-	-	0.558	0.476	0.435	0.503	0.513	0.498	0.394	0.491	0.459	0.494	0.042*	0.064	0.122	0.115	0.190*	0.392
CnaB-030	-	-	-	-	-	-	-	-	0.429	0.448	0.647	0.619	0.564	0.608	0.606	0.601	0.757	0.670	0.078*	0.098	0.243	0.229	0.400*	0.602
CnaD-112	0.726	0.648	0.686	0.607	0.636	0.641	0.512	0.523	0.714	0.724	0.682	0.719	0.615	0.682	0.606	0.602	0.730	0.690	0.615*	0.626	0.717*	0.703	0.590*	0.821
CnaF-177	0.024	0.024	0.143	0.134	0.000*	0.088	-	-	-	-	-	-	-	-	-	-	-	-	0.180*	0.265	0.292	0.338	0.050*	0.467
CtoA-247	-	-	-	-	-	-	-	-	0.416	0.399	0.388	0.396	0.282	0.281	0.364	0.327	0.270	0.240	0.181*	0.245	0.318	0.354	0.210*	0.595
CtoA-256	0.516	0.529	0.771	0.710	0.136	0.204	0.463	0.499	0.597	0.666	0.871	0.881	0.795	0.841	0.875	0.857	0.811	0.862	0.550	0.518	0.758	0.745	0.667*	0.843
CtoE-249	-	-	-	-	-	-	-	-	0.104	0.099	0.400	0.397	0.077	0.075	0.030	0.030	0.081	0.079	0.090*	0.139	0.283	0.285	0.060*	0.498
LCO3	0.258	0.305	0.214	0.277	0.250	0.282	-	-	0.416	0.526	0.753	0.734	0.718	0.708	0.688	0.699	0.730	0.671	0.181*	0.216	0.290	0.301	0.440*	0.644
Rser10	0.452	0.478	0.671	0.655	0.455	0.480	0.585	0.487	0.766	0.801	0.812	0.848	0.718	0.822	0.758	0.819	0.838	0.849	0.572	0.610	0.592	0.617	0.820*	0.823
BL1-61	0.686*	0.723	0.800	0.763	0.545	0.490	0.732	0.733	0.623	0.649	0.659	0.759	0.615	0.693	0.879	0.726	0.730	0.688	0.759*	0.792	0.785	0.833	0.720*	0.820
BL1-T2	0.629	0.651	0.671	0.673	0.614	0.557	0.634	0.581	0.740	0.727	0.694	0.717	0.590	0.592	0.485	0.661	0.649	0.695	0.627*	0.624	0.619	0.674	0.610*	0.782
Ca3	0.903	0.856	0.914	0.885	0.932	0.817	0.854	0.922	0.688	0.736	0.835	0.887	0.923	0.852	0.879	0.898	0.865	0.868	0.964	0.941	0.914	0.941	0.900	0.934
CtoF-172	0.097	0.109	0.271	0.259	-	-	0.049	0.048	0.584	0.668	0.729	0.709	0.744*	0.703	0.697	0.581	0.622	0.697	0.162*	0.191	0.311	0.324	0.460*	0.740
CypG24	0.040	0.040	0.214	0.207	-	-	0.244	0.217	0.636	0.597	0.600	0.628	0.744	0.684	0.667	0.656	0.541	0.569	0.187*	0.223	0.355	0.356	0.374*	0.750
IV04	0.339*	0.375	0.443*	0.633	0.159	0.148	0.439	0.470	0.442*	0.605	0.224	0.218	0.410	0.360	0.485	0.485	0.595	0.462	0.479	0.487	0.421	0.418	0.520*	0.537
LCO1	0.798*	0.887	0.843	0.898	0.864	0.854	0.750*	0.848	0.675	0.653	0.718	0.731	0.795	0.763	0.767	0.759	0.811	0.773	0.868*	0.925	0.962	0.927	0.750*	0.896
N7K4	0.726*	0.703	0.843	0.782	0.705	0.748	0.805	0.751	0.390*	0.465	0.306	0.328	0.615	0.629	0.636	0.596	0.622	0.668	0.743*	0.848	0.752	0.808	0.670*	0.811
Ca1	0.782	0.750	0.900	0.875	0.591	0.684	0.951	0.846	0.675*	0.818	0.788*	0.877	0.795	0.875	0.788	0.910	0.784*	0.868	0.910	0.909	0.915	0.912	0.810*	0.917
CtoG-075	-	-	-	-	-	-	-	-	0.325	0.405	0.153	0.175	0.359	0.417	0.250*	0.349	0.297	0.309	0.024*	0.041	0.093	0.091	0.280	0.310
CtoG-216	-	-	-	-	-	-	-	-	-	-	0.047	0.046	-	-	-	-	-	-	-	-	-	-	-	-
LCO5	0.048	0.047	-	-	-	-	-	-	-	-	-	-	-	-	-	-	-	-	0.102*	0.149	0.308	0.311	0.020*	0.482
Lid8	0.746	0.799	0.729	0.759	0.659	0.599	0.756	0.791	0.520*	0.673	0.529*	0.754	0.462*	0.734	0.576*	0.795	0.784	0.810	0.657*	0.723	0.589*	0.783	0.600*	0.827
Rru4	0.381*	0.501	0.500	0.582	0.477	0.485	0.561*	0.649	0.740	0.845	0.859	0.824	0.846	0.842	0.818	0.858	0.865	0.873	0.760	0.737	0.764	0.757	0.760*	0.843
Z21908	0.266	0.285	0.529	0.520	-	-	0.463	0.495	0.494*	0.773	0.526*	0.786	0.469*	0.836	0.222*	0.790	0.375*	0.809	0.442*	0.589	0.514*	0.646	0.565*	0.855
