# Supplementary material for: Non-homogeneous combination of two porous genomes induces complex body shape trajectories in cyprinid hybrids
Source: Front Zool. 2013 May 1;10:22. doi: 10.1186/1742-9994-10-22 (PMC3664599; doi:10.1186/1742-9994-10-22)
Supplement: Additional file 2 — Estimates of the frequency of null alleles for each population and marker. With k visible alleles per locus (frequencies <0.01 in italic, >0.05 in bold; “-” indicates monomophic loci). [file 1742-9994-10-22-S2.pdf]

	k	Null alleles											
		ORB(124)	SURa (70)	BER (44)	SP (41)	ALL (77)	CHE (85)	ROU (39)	MIR (33)	SURb (37)	ROS (167)	BAU (107)	JUS (100)
BL1-153	3	-	-	-	-	*0.004*	*0.000*	**0.074**	*0.000*	**0.084**	**0.052**	0.023	**0.229**
BL1-2b	7	*0.000*	*0.000*	*0.000*	0.043	*0.000*	0.040	*0.000*	**0.157**	*0.000*	0.017	*0.000*	**0.104**
BL1-30	12	0.042	*0.007*	*0.000*	*0.000*	0.017	*0.000*	*0.000*	*0.000*	*0.000*	0.028	*0.000*	*0.000*
BL1-84	9	-	*0.000*	-	-	*0.000*	*0.000*	*0.000*	*0.000*	0.024	0.038	*0.000*	**0.104**
Lce-C1	15	*0.000*	*0.000*	0.042	0.019	0.039	0.044	0.002	0.046	**0.099**	*0.000*	0.021	**0.107**
LleA-029	23	*0.000*	0.025	**0.064**	*0.000*	**0.061**	**0.059**	**0.150**	**0.059**	**0.059**	*0.003*	0.026	0.033
LleA-071	8	-	-	-	**0.057**	**0.286**	**0.154**	**0.107**	**0.080**	**0.295**	**0.093**	**0.067**	**0.284**
LleC-090	31	0.013	*0.006*	*0.002*	*0.000*	0.016	*0.000*	*0.000*	*0.004*	*0.000*	0.014	0.006	0.024
Lsou19	13	0.050	*0.000*	*0.000*	*0.000*	*0.000*	*0.000*	0.017	**0.057**	0.012	*0.007*	*0.000*	**0.075**
BL1-98	10	**0.057**	**0.067**	*0.000*	*0.000*	0.015	*0.001*	*0.000*	*0.000*	*0.000*	0.030	0.042	0.011
BL2-114	8	*0.000*	*0.000*	*0.000*	-	*0.000*	0.022	*0.000*	*0.000*	*0.000*	**0.063**	**0.081**	**0.109**
LceA-149	3	-	*0.000*	-	-	-	-	-	-	-	**0.077**	0.048	**0.302**
LleA-150	36	*0.000*	**0.083**	**0.053**	*0.000*	*0.000*	**0.097**	*0.003*	*0.000*	0.046	0.014	0.038	0.013
Lsou05	13	*0.000*	*0.000*	*0.000*	**0.052**	*0.000*	*0.000*	0.046	*0.000*	*0.000*	0.017	0.027	0.018
Lsou08	10	*0.000*	*0.000*	*0.000*	*0.000*	*0.000*	*0.000*	*0.000*	**0.070**	*0.005*	0.026	*0.000*	**0.097**
Lsou29	29	*0.002*	*0.005*	0.013	*0.000*	0.016	**0.060**	**0.108**	**0.098**	0.030	0.032	*0.000*	0.043
Lsou34	10	0.018	*0.000*	*0.000*	*0.000*	**0.091**	**0.170**	**0.178**	**0.158**	**0.114**	*0.000*	**0.063**	**0.172**
Ppro132	2	-	-	-	-	*0.000*	0.043	*0.000*	**0.061**	0.019	**0.059**	*0.000*	**0.157**
CnaB-030	3	-	-	-	-	*0.006*	*0.000*	0.031	*0.000*	*0.000*	**0.051**	*0.000*	**0.116**
CnaD-112	9	*0.000*	*0.000*	0.011	*0.000*	*0.004*	0.023	0.035	*0.000*	*0.000*	0.028	0.017	**0.120**
CnaF-177	3	*0.000*	*0.000*	**0.143**	-	-	-	-	-	-	**0.091**	**0.060**	**0.287**
CtoA-247	5	-	-	-	-	*0.000*	*0.000*	*0.000*	*0.000*	*0.000*	**0.098**	0.044	**0.237**
CtoA-256	15	*0.000*	*0.000*	**0.084**	*0.004*	0.026	*0.000*	0.012	*0.000*	0.036	*0.000*	0.028	**0.099**
CtoE-249	3	-	-	-	-	*0.000*	*0.000*	*0.000*	*0.000*	*0.000*	**0.081**	*0.000*	**0.293**
LCO3	6	0.047	**0.065**	0.022	-	**0.075**	*0.000*	*0.000*	0.011	*0.000*	**0.065**	*0.006*	**0.115**
Rser10	10	*0.004*	*0.000*	0.014	*0.000*	0.021	0.023	**0.056**	**0.053**	*0.000*	0.022	*0.000*	*0.002*
BL1-61	9	0.029	*0.000*	*0.000*	0.018	*0.000*	**0.052**	0.030	*0.000*	*0.000*	0.012	0.014	0.044
BL1-T2	10	*0.009*	*0.000*	*0.000*	*0.000*	*0.000*	*0.000*	*0.000*	**0.107**	*0.000*	0.023	0.032	**0.078**
Ca3	32	*0.000*	*0.000*	*0.000*	0.040	0.034	0.027	*0.000*	*0.000*	*0.000*	*0.000*	0.012	0.013
CtoF-172	7	0.035	*0.000*	-	*0.000*	0.037	*0.000*	*0.000*	*0.000*	**0.051**	0.012	*0.000*	**0.144**
CypG24	10	*0.000*	*0.000*	-	*0.000*	*0.000*	*0.000*	*0.000*	*0.000*	*0.000*	**0.074**	*0.000*	**0.215**
IV04	4	**0.054**	**0.127**	*0.000*	0.018	**0.103**	*0.000*	*0.000*	*0.000*	*0.000*	0.020	*0.000*	0.035
LCO1	34	0.035	0.016	*0.000*	0.042	*0.000*	0.003	*0.000*	0.035	*0.000*	0.026	*0.000*	**0.069**
N7K4	15	0.019	*0.000*	*0.009*	*0.000*	**0.064**	0.014	*0.000*	*0.000*	0.023	0.048	0.014	**0.063**
Ca1	37	*0.000*	0.011	0.050	*0.000*	**0.068**	0.045	0.037	**0.054**	**0.054**	*0.000*	*0.000*	**0.055**
CtoG-075	3	-	-	-	-	**0.062**	0.023	0.026	**0.141**	0.026	0.046	*0.000*	0.019
CtoG-216	2	-	-	-	-	-	*0.000*	-	-	-	-	-	-
LCO5	2	*0.000*	-	-	-	-	-	-	-	-	**0.075**	*0.001*	**0.313**
Lid8	34	0.017	*0.000*	*0.000*	0.011	**0.098**	**0.132**	**0.144**	**0.128**	0.025	0.041	**0.087**	**0.121**
Rru4	22	**0.125**	0.048	*0.001*	0.021	0.048	*0.000*	*0.000*	*0.000*	*0.005*	*0.000*	0.014	0.030
Z21908	16	0.028	0.015	-	0.018	**0.156**	**0.201**	**0.276**	**0.463**	**0.360**	**0.123**	**0.118**	**0.205**
