# Supplementary figures and images for: Non-homogeneous combination of two porous genomes induces complex body shape trajectories in cyprinid hybrids
Source: Front Zool. 2013 May 1;10:22. doi: 10.1186/1742-9994-10-22 (PMC3664599; doi:10.1186/1742-9994-10-22)

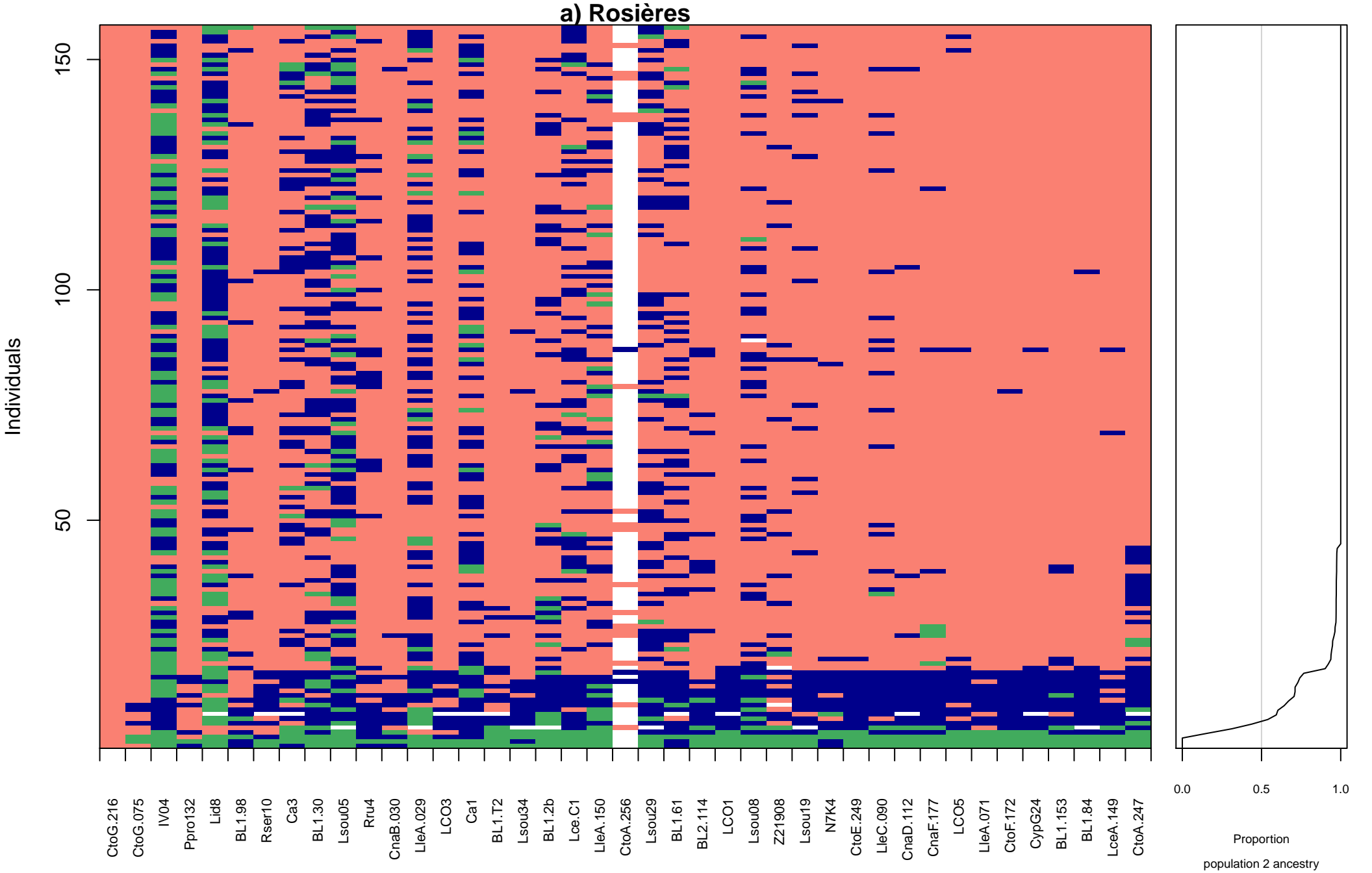

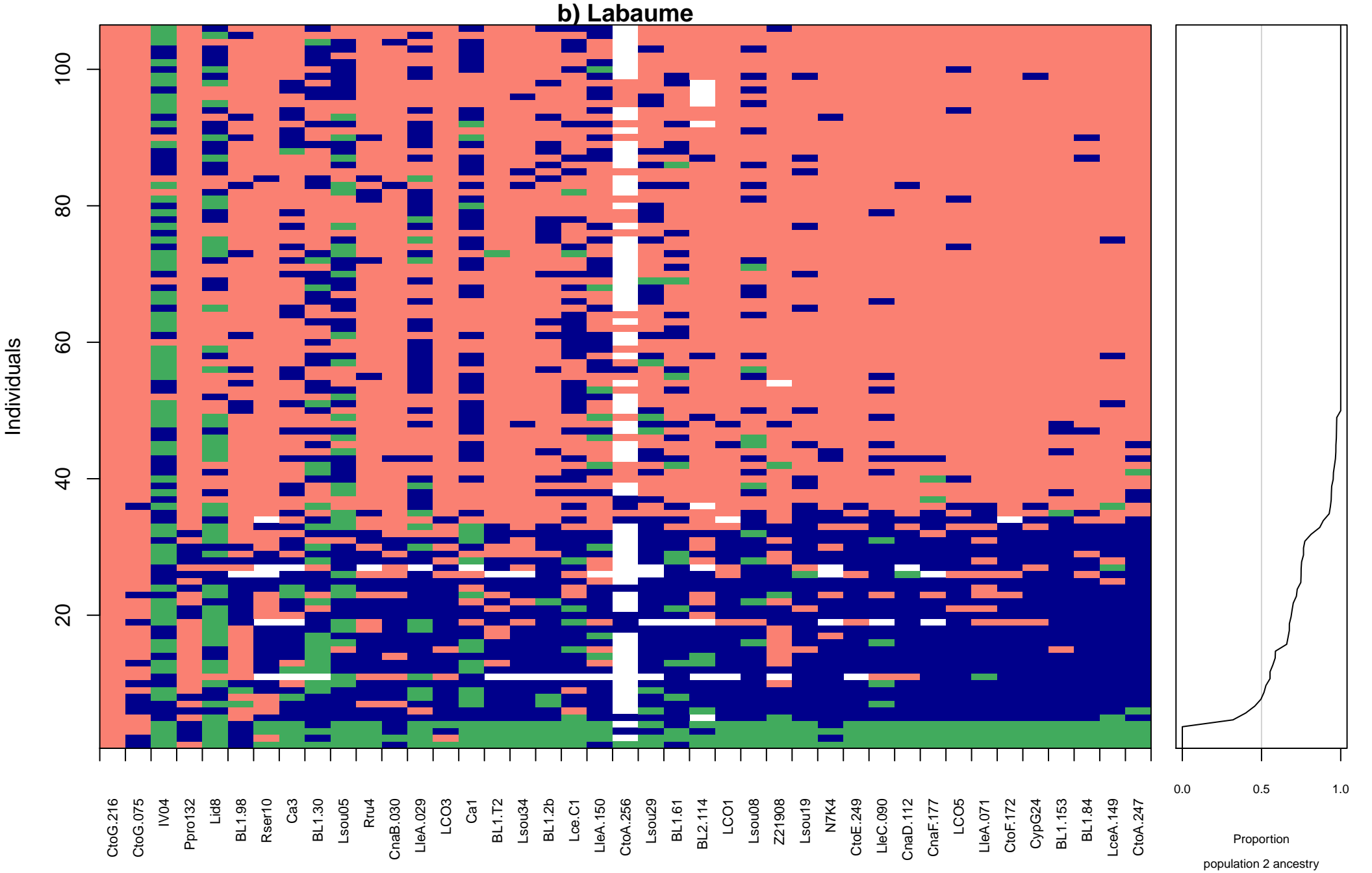

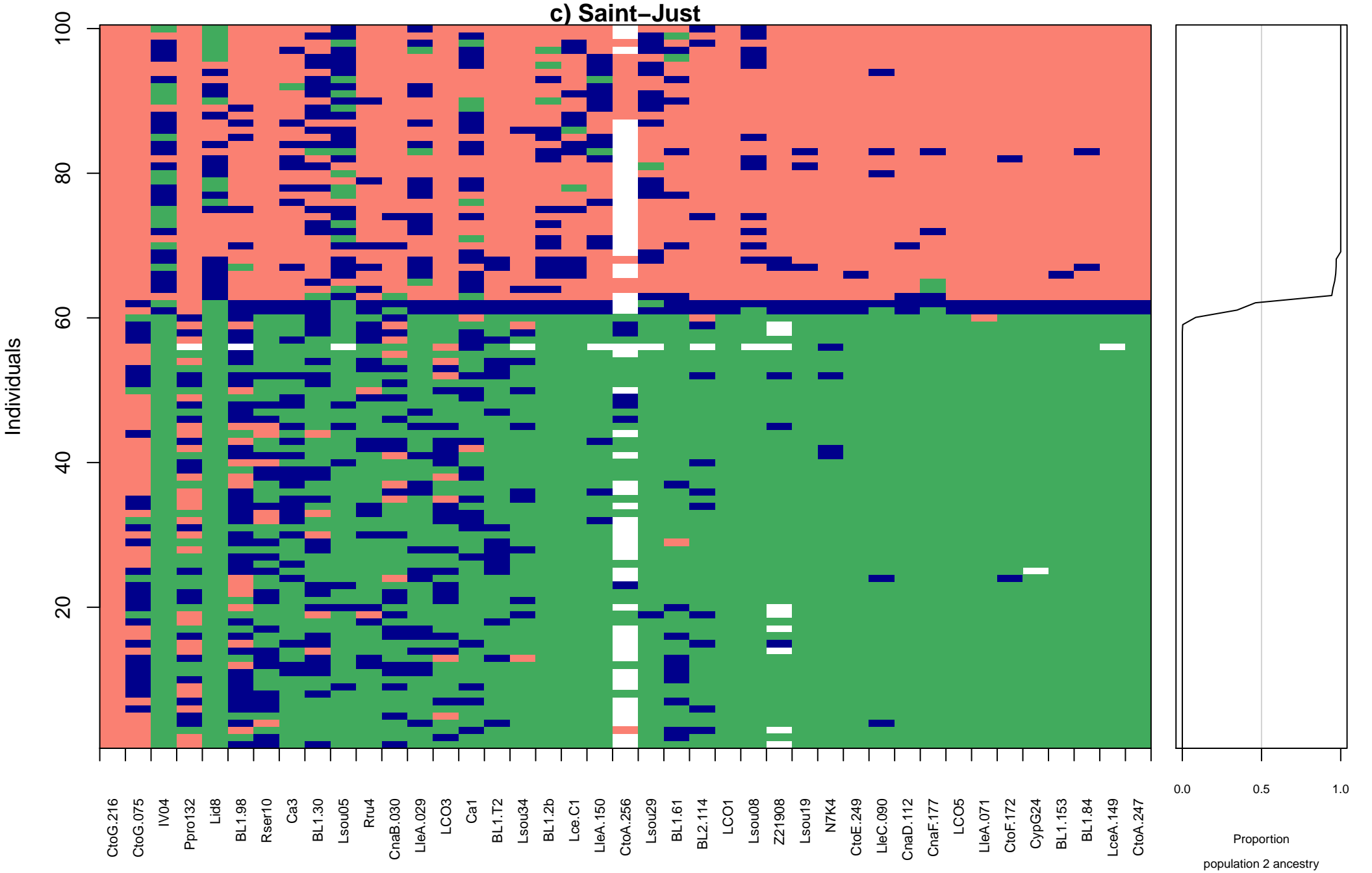

Supplement: Additional file 3 — Assignment of individuals withIntrogressand estimation of h-index. a) for Rosières b) for Labeaume c) for Saint-Just. (green: h = 0 corresponding to C. nasus assignment, blue: h between 0 and 1 corresponding to hybrid assignment, and pink: h = 1 corresponding to P. toxostoma assignment). [file 1742-9994-10-22-S3.pdf]

a)

K= 2

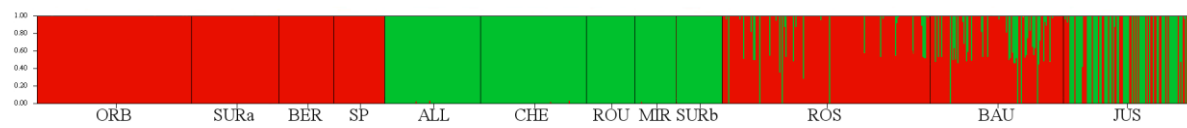

K = 3

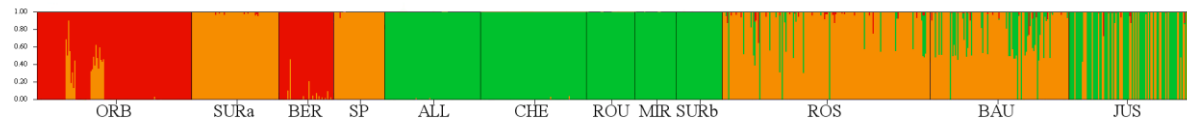

K = 4

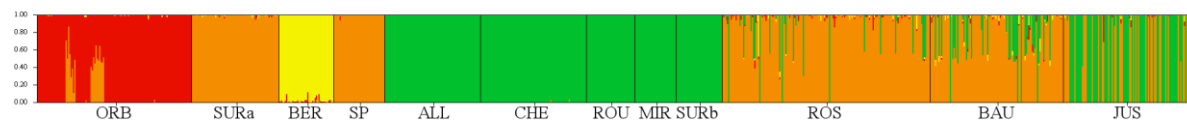

K = 5

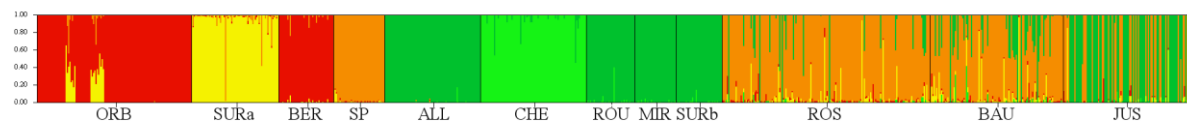

K = 6

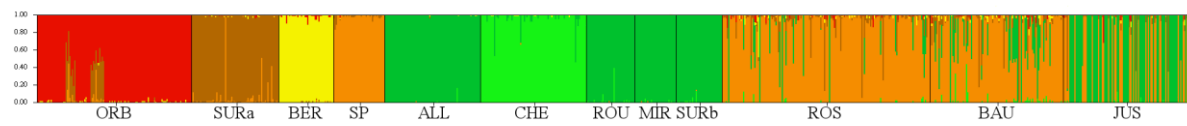

b)

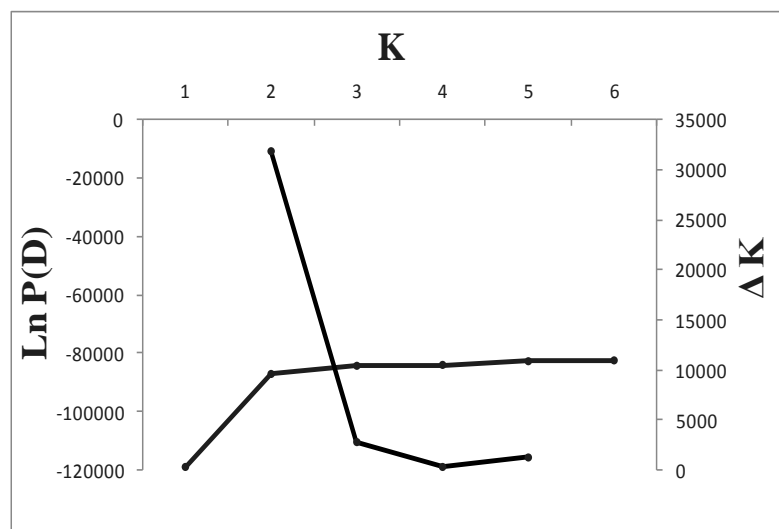

Supplement: Additional file 4 — Admixture withStructuresoftware between populations ofP. toxostomaandC. nasus. a) Graphs of admixtures of populations from K = 2 to K = 6. b) Posterior probability (LnP(d)) and ΔK as a functions of K group. [file 1742-9994-10-22-S4.pdf]

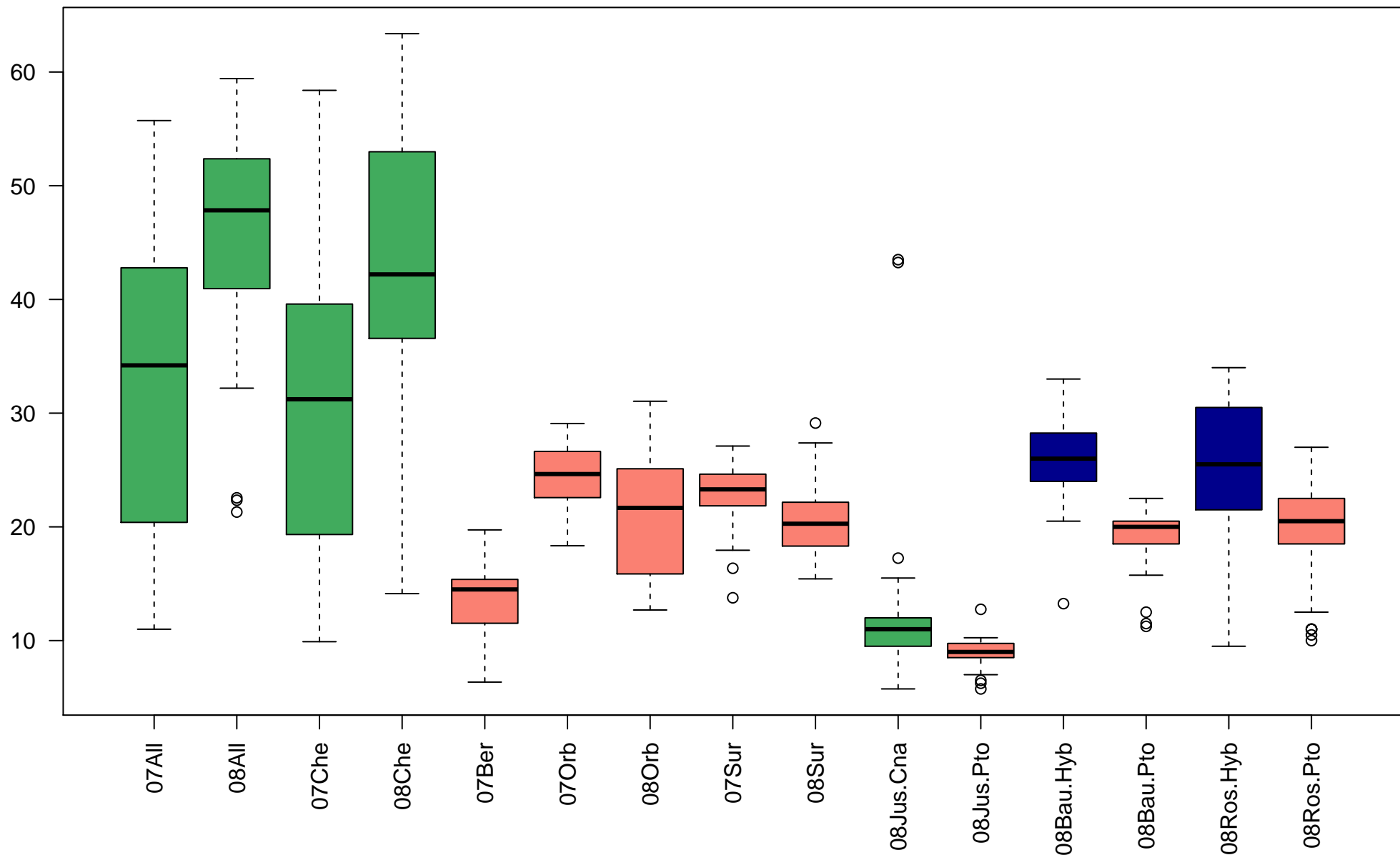

Supplement: Additional file 6 — Box-plot of size for each reference population and Ardèche population for each year. (in green C. nasus, in red P. toxostoma and in blue hybrids). These individuals are those considered in the DA. [file 1742-9994-10-22-S6.pdf]

**Ardèche r = 0.76 P = 0**

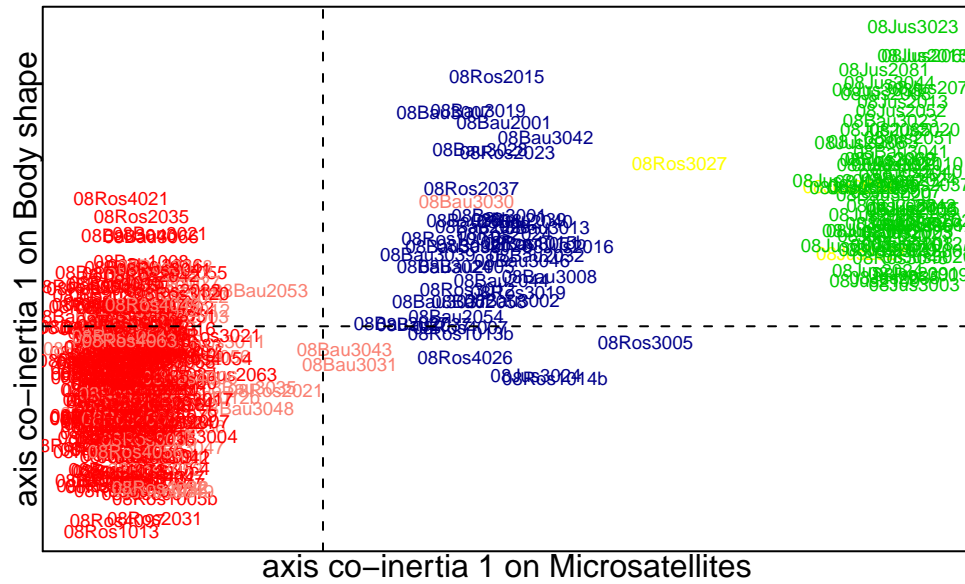

**Saint-Just r = 0.88 P = 0**

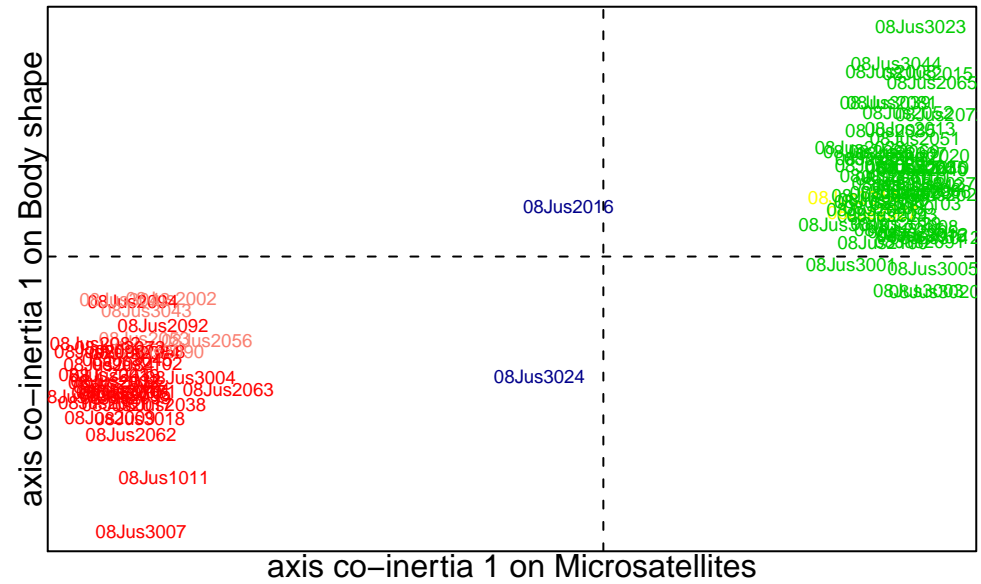

**Labauve r = 0.82 P = 0**

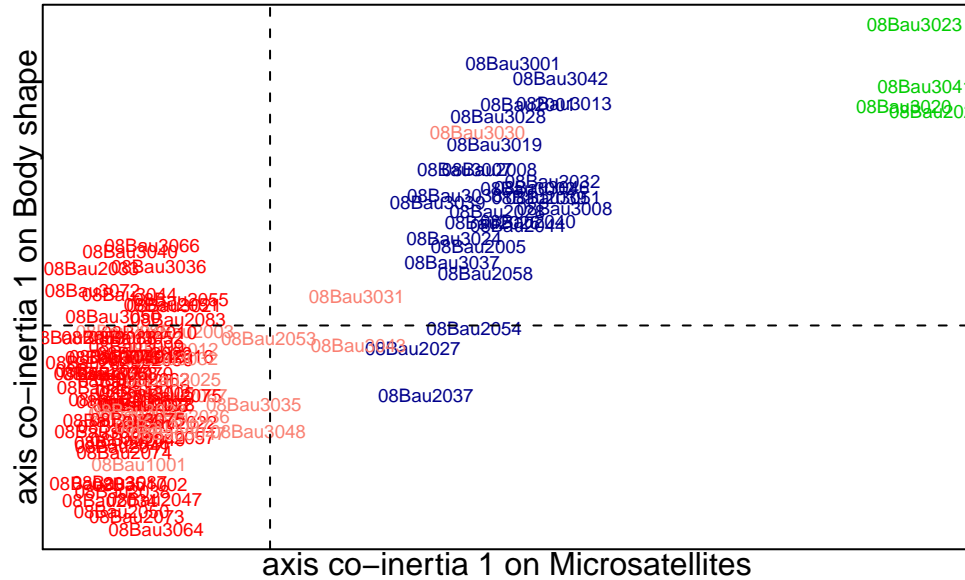

**Rosières r = 0.66 P = 0**

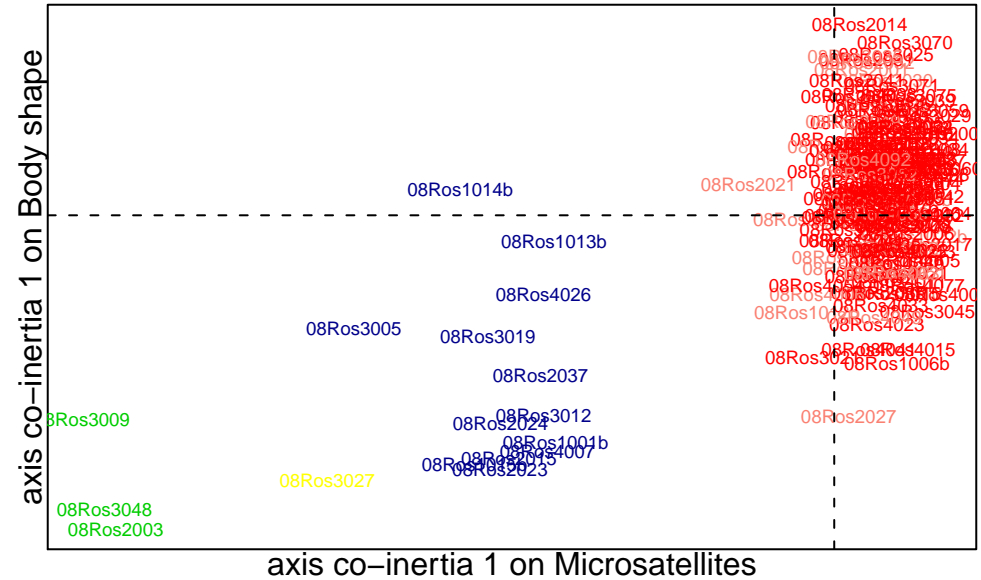

Supplement: Additional file 7 — Co-inertia analysis. Plot of first co-inertia analysis axis for body shape as a function of first co-inertia analysis for microsatellites (green: h = 0, yellow: 0 < h < 0.3, blue: h between 0.3 and 0.8, pink: 0.8 < h < 1, red: h = 1). [file 1742-9994-10-22-S7.pdf]
