# Supplementary material for: Non-homogeneous combination of two porous genomes induces complex body shape trajectories in cyprinid hybrids
Source: Front Zool. 2013 May 1;10:22. doi: 10.1186/1742-9994-10-22 (PMC3664599; doi:10.1186/1742-9994-10-22)
Supplement: Additional file 5 — Determination of marker genomic cline for each population of the Ardèche and populations without F1 individuals. (DS+: positive directional selection of invasive C. nasus homozygotes; DS-: negative directional selection of C. nasus homozygotes; OD: overdominance for interspecific heterozygotes; UD: underdominance for interspecific heterozygotes; EP: epistasis; IA: increase admixture; M: monomorph; “-” indicates neutral loci). [file 1742-9994-10-22-S5.pdf]

	Ros	Ros.wF1	Bau	Bau.wF1	Jus	Jus.wF1
BL1-153	-	-	-	-	-	-
BL1-2b	-	-	OD	OD/DS-	-	-
BL1-30	DS+	DS+	UD	DS+	-	-
BL1-84	OD	OD	OD	-	-	-
Lce-C1	-	-	-	-	-	-
LleA-029	DS+	DS+	DS+	DS+	-	-
LleA-071	EP	EP	-	-	-	UD
LleC-090	-	-	-	-	-	-
Lsou19	OD/DS-	-	-	-	-	-
BL1-98	-	-	-	-	IA	S
BL2-114	-	-	-	-	-	-
LceA-149	-	-	-	-	-	-
LleA-150	UD/DS+	UD/DS+	IA	DS+	-	-
Lsou05	DS+	DS+	IA	DS+	DS+	DS+
Lsou08	-	-	-	-	-	-
Lsou29	DS+	DS+	UD	-	DS+	S
Lsou34	-	-	-	-	-	-
Ppro132	-	-	UD	-	-	-
CnaB-030	-	-	-	-	-	-
CnaD-112	-	-	-	-	-	-
CnaF-177	IA	IA	-	-	-	-
CtoA-247	DS+	-	-	-	-	-
CtoA-256	-	-	OD/DS+	OD	-	-
CtoE-249	OD	OD	OD	OD	-	-
LCO3	DS+	S	-	-	-	-
Rser10	-	-	-	-	-	-
BL1-61	IA	IA	IA	IA	-	-
BL1-T2	-	-	-	-	-	-
Ca3	-	-	-	-	-	-
CtoF-172	OD	OD	OD/DS-	OD/EP	-	-
CypG24	OD	OD	OD	-	-	-
IV04	-	-	-	-	-	S
LCO1	OD/EP	OD/EP	OD/DS-	OD/EP	EP	UD
N7K4	-	-	-	-	-	-
Ca1	DS+	DS+	DS+	UD/DS+	DS+	DS+
CtoG-075	UD	UD	-	-	-	S
CtoG-216	M	M	M	M	M	M
LCO5	-	-	-	-	-	-
Lid8	UD/DS+	UD/DS+	UD/DS+	UD/DS+	-	-
Rru4	-	-	-	-	-	-
Z21908	-	-	-	-	-	-
